# Conversion of Natural Narciclasine to Its C-1 and C-6 Derivatives and Their Antitumor Activity Evaluation: Some Unusual Chemistry of Narciclasine †

**DOI:** 10.3390/molecules27134141

**Published:** 2022-06-28

**Authors:** Juana Goulart Stollmaier, Jared Thomson, Mary Ann Endoma-Arias, Razvan Simionescu, Alexandra Vernaza, Nakya Mesa-Diaz, Mitchell Smith, Liqin Du, Alexander Kornienko, Tomas Hudlicky

**Affiliations:** 1 Department of Chemistry, Brock University, 1812 Sir Isaac Brock Way, St. Catharines, ON L2S 3A1, Canada; jt15gq@brocku.ca (J.T.); maryann.arias@bpt.eurofinsca.com (M.A.E.-A.); rsimionescu@brocku.ca (R.S.); thudlicky@brocku.ca (T.H.); 2 Department of Chemistry and Biochemistry, Texas State University, San Marcos, TX 78666, USA; a_v651@txstate.edu (A.V.); nlm59@txstate.edu (N.M.-D.); mts85@txstate.edu (M.S.); l_d141@txstate.edu (L.D.)

**Keywords:** narciclasine, *Amaryllidaceae* alkaloids, Semi-synthesis, natural products

## Abstract

During the search for a general, efficient route toward the synthesis of C-1 analogues of narciclasine, natural narciclasine was protected and converted to its C-1 enol derivative using a novel semi-synthetic route. Attempted conversion of this material to its triflate in order to conduct cross-coupling at C-1 resulted in a triflate at C-6 that was successfully coupled with several functionalities. Four novel compounds were fully deprotected after seven steps and subjected to evaluation for cytotoxic activity against three cancer cell lines. Only one derivative showed moderate activity compared to that of narciclasine. Spectral and physical data are provided for all new compounds.

## 1. Introduction

**Figure 1:**
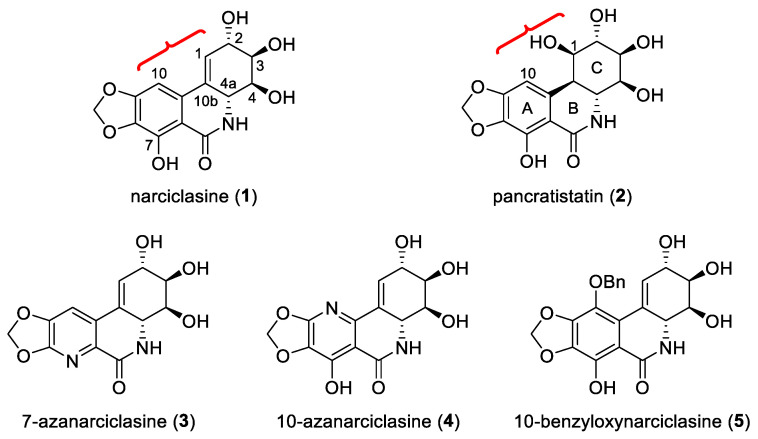
Major Amaryllidaceae constituents and unnatural derivatives of narciclasine.

Narciclasine (**1**) and pancratistatin (**2**) (Figure 1), have been among the most studied constituents of the *Amaryllidaceae* family of alkaloids since their isolation from *Narcissus* bulbs in 1967 [1], and *Pancratium littorale* in 1984 [2,3], respectively. Their unique antitumor activity has led not only to many synthetic approaches, but also to investigations of unnatural derivatives with a focus on providing more bioavailable compounds. The syntheses of these natural products and their unnatural derivatives, and the study of their biological activities have been reviewed on many occasions, with the last major reports published in 2016 and 2017 [4,5,6,7,8,9,10,11,12,13,14,15]. Synthetic activity continued, and further creative approaches to both **1** and **2** and their related congeners have been reported since [16,17,18,19].

The minimum pharmacophore for the Amaryllidaceae alkaloids has been suggested [20], and changes in structure are allowed in the “northwest bay region,” the space outside C-1 and C-10 indicated in Figure 1, without detriment to biological activity. Many derivatives of pancratistatin have been made and tested [21,22,23,24,25,26]. Some of the unnatural derivatives showed enhanced biological activities, namely the pancratistatin C-1 benzoate reported by Pettit [27] and the C-1 benzoyloxymethyl compounds reported by us [28,29] (both with nanomolar activity). These findings provided further impetus to continue this research with pancratistatin as well as narciclasine. There are not as many unnatural derivatives reported for the latter alkaloid compared to the research volume with pancratistatin. We have published several fully synthetic approaches to C-7 and C-10 analogues, namely compounds **3**, **4**, and **5**, shown in Figure 1 [30,31,32,33]. Only one of these, 10-azanarciclasine (**4**), displayed activity comparable to the natural product.

The preparation of unnatural derivatives of pancratistatin or narciclasine is arduous and generally requires a unique approach for each derivative. For this reason, we have recently embarked on a program seeking to convert natural narciclasine, available by extraction from daffodil bulbs, to its C-1 derivatives. In this paper, we report the synthesis of several derivatives prepared by cross-coupling, along with the interesting and unique reactivity of narciclasine. The results of initial biological evaluations are provided for the new derivatives.

## 2. Results and Discussion

### 2.1. Initial Approach to the Synthesis of C-1 Analogues

**Scheme 1:**
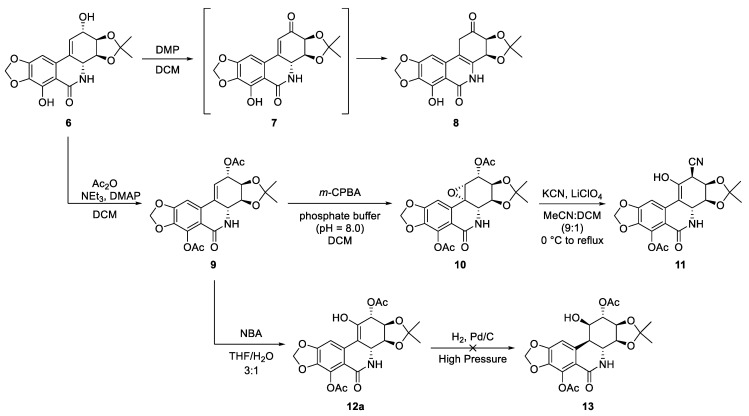
Originally planned routes to C-1 analogues. Reagents and solvents abbreviations: Dess-Martin Periodinane (DMP); Dichloromethane (DCM), Acetic anhydride (Ac_2_O), *meta*-chloroperoxybenzoic acid (*m*-CBPA)*,* Triethylamine (NEt_3_), 4-Dimethylaminopyridine (DMAP), Acetonitrile (MeCN), *N*-bromoacetamide (NBA), Tetrahydrofuran (THF).

We initiated our approach to C-1 analogues by pursuing established chemistry to functionalize the C-1 position (Scheme 1). The first attempt involved oxidation of protected narciclasine to the corresponding enone **7**. We expected to be able to prepare the C-1 vinyl bromide for cross-coupling by bromination, followed by a dehydrobromination process. This approach was met with failure because of the instability of **7** and its isomerization to enamide **8**.

Epoxidation of narciclasine, as previously reported by Pettit [27], seemed to be another viable option for functionalizing the C-1 position. Initial attempts to open epoxide **10** with various nucleophiles proved to be a more difficult task than originally accounted for. Epoxide **10** was inert to several conditions, and upon treatment with KCN, the reaction yielded a compound that was later identified as a C-1 enol C-2 nitrile (**11**).

When trying to form the *syn*-epoxide with *N*-bromoacetamide (NBA), the reaction afforded the C-1 enol **12a** instead of the desired bromohydrin intermediate [34]. Hydrogenation of this intermediate was attempted as it would provide a short conversion of narciclasine to pancratistatin. The enol, or, more accurately, the vinylogous phenol, did not undergo hydrogenation even under high pressure (500 psi). The formation of the stable enol was both unusual and surprising, but it provided the means for potential cross-coupling through pseudohalide groups (either tosylate or triflate) at C-1, and we have focused on this particular strategy.

**Scheme 2:**
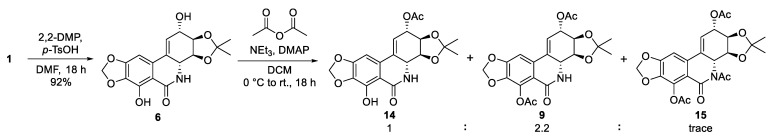
Initial protecting group steps. Reagents and solvents abbreviations: 2,2-Dimethoxypropane (2,2-DMP), *p*-Toluenesulfonic acid (*p*-TsOH), Dimethylformamide (DMF).

**Scheme 3:**
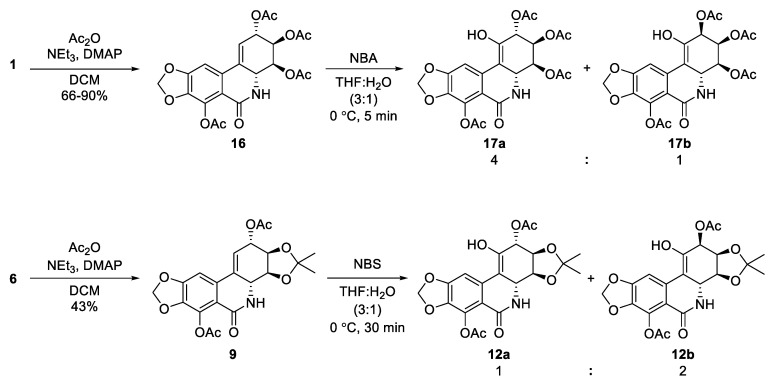
Main routes for the synthesis of C-1 enol and its C-2 epimer. Reagents and solvents abbreviations: *N*-bromoacetamide (NBA), *N*-bromosuccinimide (NBS).

Narciclasine (**1**) was protected as an acetonide at C-3/C-4, and the C-2/C-7 hydroxyl groups were acylated to furnish diacetate **9** along with monoacetate **14** in a ratio of 2.2:1, respectively, and trace amounts of triacetate **15** (Scheme 2). In addition, narciclasine was also converted to its peracetate **16** (Scheme 3) in order to compare the steric influence of the acetonide group versus acetates on the chemical events taking place at C-1.

**Figure 2:**
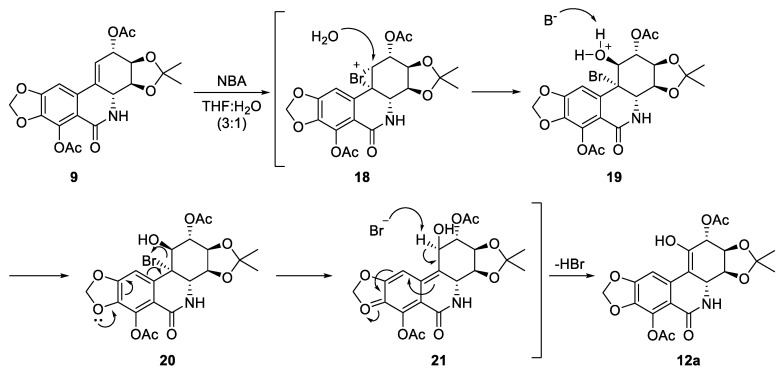
Suggested mechanism for the formation of C-1 enol.

Treatment of either narciclasine peracetate (**16**) or narciclasine diacetate (**9**) with NBA (or NBS) provided the C-1 enols **17a** and **12a**, respectively, along with the C-2 epimers **17b** and **12b**, as shown in Scheme 3. It is believed that this reaction proceeds by the formation of a bromonium species and its opening by water at the C-1 position. Elimination of the benzylic bromine follows, yielding the C-1 enol (Figure 2). This is accompanied by the partial epimerization at C-2 through keto-enol tautomerization, facilitated by the generation of HBr and the resulting acidic medium. The suggested mechanism for this transformation is shown in Figure 2. The ratio of the corresponding C-2 epimers appears to depend on the reaction time and the protecting groups on the C-ring. The ratio of enols **17a**/**17b** was 4:1 after five minutes, while the ratio of **12a**/**12b** was 1:2 after 30 min. The reaction proceeds almost instantly and can be quenched within minutes. The faster it is quenched, the lesser the amount of C-2 epimer observed.

### 2.2. Synthesis of Cross-Coupling Substrates

**Scheme 4:**
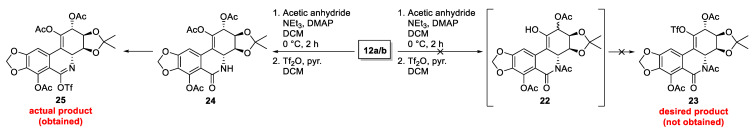
The unexpected synthesis of C-6 triflate from C-1 enol narciclasine diacetate **12a**/**b**.

With the enols **12ab**, **17ab** in hand, we set out to prepare the C-1 triflate and subjected the resulting compounds to cross-coupling. However, early trials resulted in sluggish reactions with low yields of the desired vinyl triflate. It was believed that such results were observed because of potential competition between the C-1 enol and the ring-B lactam. The mixture of C-1 enols **12a** and **12b** was treated with acetic anhydride in order to obtain imide **22** that would then be converted to the C-1 triflate (Scheme 4). Surprisingly, upon treating together the C-2 epimers **12a** and **12b** with acetic anhydride, the mixture equilibrated under these conditions and produced the enol acetate **24** with the correct C-2 stereochemistry rather than the expected diastereomeric mixture of imide **22**. The enol acetate **24** was erroneously further functionalized under triflation conditions, as shown on Scheme 4.

Three initial cross-coupling partners were selected (phenylboronic acid, phenyl acetylene and potassium vinyl trifluoroborate), and cross-coupling reactions were further performed on what was believed to be “triflate” **23**, later determined to be **25**. “Triflate” **23** reacted with moderate yields with all three reagents.

During further investigation of the reactivity of the C-1 triflate **23** and in the cross-coupling reactions, we noticed that the experimental data of the expected imide **22** and the product of a Prévost reaction of diacetate **9** were identical. This fact cast serious doubt on the identity of the C-1 cross-coupled products. From ^1^H-^15^N-NMR HSQC and HMBC experiments, it was determined that the imide **22** had been misassigned, and it is in fact acetate **24**. The structure of enol acetate **24** is consistent with the experimental data and would explain how the stereochemistry at C-2 has been “fixed” and “locked”, since the presence of an enol acetate would prevent any further keto-enol tautomerization and the epimerization at C-2, which is only possible while the C-1 functionality is a free enol. We therefore had to re-evaluate the structures of the expected cross-coupling substrate **23**. 

Additional ^1^H-^15^N-NMR HSQC and HMBC experiments were performed on **23** and showed the absence of -NH and a large change in δ_N_ chemical shift from 150 ppm (amide) to 315 ppm (pyridine-like nitrogen atom). Upon inspection of IR, the characteristic band for lactams was absent (~1670 cm^−1^). The cross-coupling conditions (Sonogashira, Suzuki) were still working, although in low to moderate yield. We noted that C-N cross-couplings of that nature have not been reported in the literature and that such a scenario would not fit in Buchwald-Hartwig cross-coupling conditions [35,36]. Based on these additional data, we have proposed that the cross-couplings occurred at the C-6 position of triflate **25** (Scheme 4).

**Figure 3:**
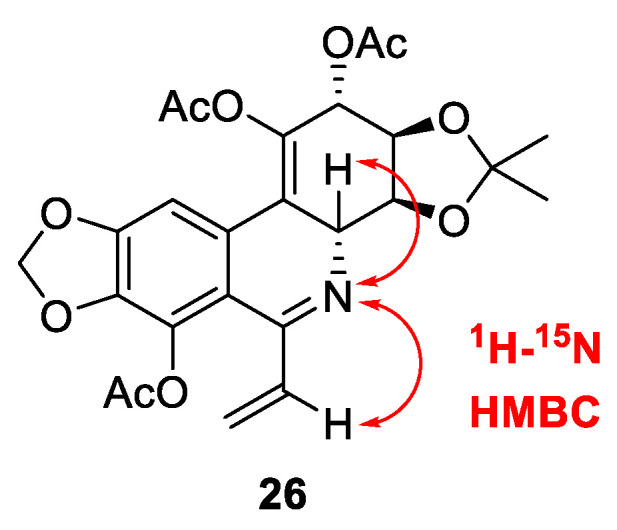
Structure of C-6 vinyl **26** and detected ^1^H-^15^N-NMR correlations highlighted by red arrows.

The synthesis of the vinyl cross-coupling product (**26**) provided more data useful to elucidate the cross-coupling process at the C-6 position. Upon analysis with ^1^H-^15^N-NMR HMBC, two correlation signals were observed: N-H (4a) and N-H (vinyl) (Figure 3). 

A correlation signal between the vinylic proton and nitrogen could only logically occur in two situations: if the vinyl group was directly bound to nitrogen, or if it was at C-6. The latter scenario is the most likely to be the correct one as the coupling constants agree with the structure. After such findings, further investigations of the structure of the fully deprotected products obtained from the cross-couplings performed on the C-6 triflate was thus required. It is important to note the lack of a carbonyl peak from ^13^C-NMR in the region around 169 ppm on the fully deprotected products. This region is where the lactam carbonyl peak of narciclasine is expected. 

**Scheme 5:**
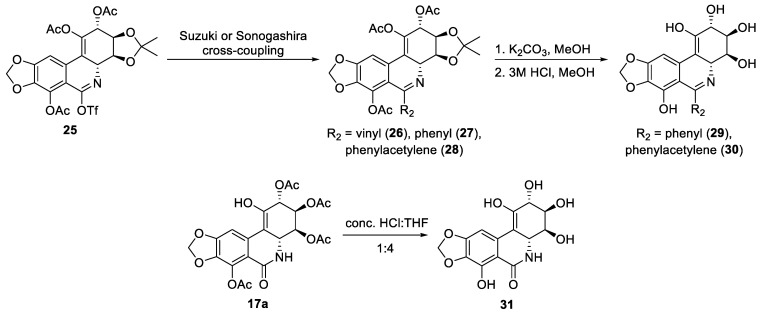
General strategy for the global deprotection of the C-6 derivatives **26**–**28** and C-1 enol **17a**.

Deprotection of the C-1 enol **17a**, as well as the C-6 derivatives, was relatively straightforward. In the case of **17a**, treatment with NaOMe in methanol did not promote the formation of product **31**, so it was decided to use concentrated HCl in THF. After quenching the reaction with solid NaHCO_3,_ it yielded the desired deprotected product **31**. In the case of the C-6 derivatives, treatment with potassium carbonate in methanol followed immediately by treatment with 3 M HCl gave the desired deprotected products, as shown in Scheme 5. 

**Scheme 6:**
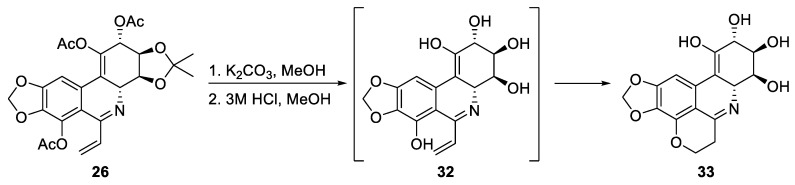
Unexpected cyclization of 26 under deprotection conditions.

However, in the case of compound **26**, the standard conditions shown in Scheme 5 promoted cyclization of the vinyl group (Scheme 6). It is believed that the deprotection of **26** produces **32**, an α,β-unsaturated imine that could undergo intramolecular Michael addition with the phenol to yield dihydropyran **33**. 

**Figure 4:**
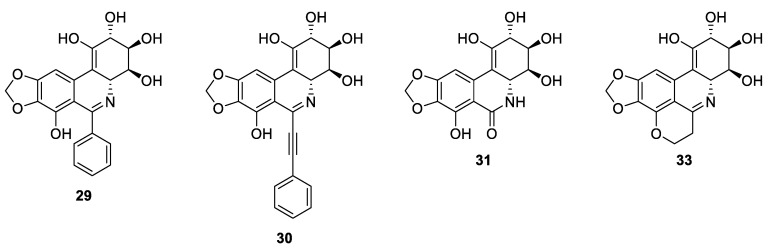
Structures of biologically evaluated C-1/C-6 narciclasine analogues.

The four deprotected compounds **29**, **30**, **31** and **33** (Figure 4), together with narciclasine (**1**) and pancratistatin (**2**) controls, were subjected to biological evaluation. The results are shown in Table 1.

We used three in vitro cancer models—neuroblastoma BE(2)-C, lung squamous cell carcinoma H157 and lung adenocarcinoma cells A549 (Table 1, Supplementary Figure S1). As expected [33], the narciclasine and pancratistatin controls showed good nanomolar activity against all cancer cell lines. Out of the synthesized compounds, only phenyl derivative **29** showed moderate single digit micromolar activity against BE(2)-C and H157 cells. Phenylacetylene derivative **30** also displayed activity, albeit of significantly diminished potency, and most likely through a different mode of action due to marked structural differences. Surprisingly, enol **31** failed to register any pronounced activity against any of the cell lines. Although structurally, **31** resembles both **1** and **2**, the stability of such enol functionality in a biological medium is an important consideration. Overall, the B-ring lactam appears to be an important part of the cytotoxic pharmacophore and the removal of the lactamic carbonyl has a deleterious effect on activity, although it does not abolish it altogether.

## 3. Materials and Methods

All solvents were distilled and kept dry before usage. Unless otherwise stated, all reactions were done in an inert atmosphere (Ar or N_2_). All reagents were obtained from commercial sources. Nuclear magnetic resonance (NMR) analyses were performed on Bruker Avance AV 300, Bruker Avance III HD 400 and Bruker Avance AV 600 digital NMR spectrometers, running Topspin 2.1 and 3.5 software. The probes are furnished with VT (variable temperature) and gradient equipment. Chemical shifts are given in δ, relative integral, multiplicity (singlet (s), doublet (d), triplet (t), quartet (q), multiplet (m)) and coupling constants (*J*) in Hz. Melting points (m.p.) were measured in a capillary apparatus. Mass spectra (HRMS) measurements were determined on an LTQ Orbitrap XL. The molecular mass-associated ion was measured by electron ionization, electrospray ionization or fast atom bombardment. Infrared (IR) spectra were recorded on an FT-IR spectrophotometer as neat and are reported in wave numbers (cm^−1^) and intensity (broad (br), strong (s), medium (m), weak (w)). Column chromatography was performed on flash grade 60 silica gel. Thin-layer chromatography (TLC) was performed on silica gel 60 F254-coated aluminum sheets. TLC plates were visualized using UV and stained with iodine, cerium ammonium molybdate (CAM), KMnO_4_ solutions, FeCl_3_ solutions, ninhydrin solutions or 2,4-dinitrophenylhydrazine (2,4-DNP) solutions.

(3a*S*,3b*R*,12*S*,12a*R*)-6,12-dihydroxy-2,2-dimethyl-3b,4,12,12a-tetrahydrobis([1,3]dioxolo)[4,5-c:4′,5′-j]phenanthridin-5(3aH)-one (**6**) [37].

Narciclasine **1** (515 mg, 1.67 mmol) was solubilized in DMF (5 mL) under inert conditions, and 2,2-DMP (1 mL, 8.16 mmol) was added, followed by a catalytic amount of p-TSA. The solution was left stirring overnight at room temperature, and on the next day, a white precipitate was present. The reaction was quenched with pyridine (1 mL, 12.4 mmol) and water (5 mL), left it stirring for one extra hour and the mixture was filtered through vacuum filtration. Acetonide-narciclasine **6** (530 mg, 1.53 mmol, 91% yield) was collected as a white solid and used without further purification.

**6**: $\alpha_{D}^{22}=-30.16\;\left(c=0.45,\mathrm{DMF}\right),\;$lit. [37]$\;\alpha_{D}^{20}=-33\;\left(c=0.35,\;\mathrm{THF}\right);$ m.p. 270 °C (from DMF) (decomposition), lit. [12] 270–274 °C (from DMF); ^1^H-NMR (300 MHz, DMSO) δ 13.74 (s, 1H), 7.00 (s, 1H), 6.47 (t, J = 2.9 Hz, 1H), 6.12–6.03 (m, 2H), 5.81 (d, J = 5.7 Hz, 1H), 4.16 (td, J = 5.7, 2.9 Hz, 1H), 4.13–4.02 (m, 2H), 3.97 (dd, J = 7.3, 5.9 Hz, 1H), 1.46 (s, 3H), 1.32 (s, 3H); ^13^C-NMR (75 MHz, DMSO) δ 167.65, 152.55, 145.22, 133.35, 128.89, 128.25, 125.93, 109.83, 104.27, 102.05, 94.22, 79.01, 78.46, 71.03, 54.63, 27.07, 24.80; LRMS (EI) *m/z* 347.10 (M^+^, 23%), 271.06 (25), 247.09 (base peak), 242.09 (41), 157.11 (23), 57.64 (24); HRMS (EI) calculated for C_17_H_17_NO_7_ 347.1005; found 347.1002. The spectral data were matched and are in agreement with the literature data [37].

(3a*S*,3b*R*,12*S*,12a*R*)-2,2-dimethyl-5-oxo-3a,3b,4,5,12,12a-hexahydrobis([1,3]dioxolo)[4,5-c:4′,5′-j]phenanthridine-6,12-diyl diacetate (**9**) [37].

Acetonide-narciclasine **6** (460 mg, 1.32 mmol) was flushed with the Schlenk technique and suspended in DCM (10 mL). The reaction mixture was cooled to 0 °C in an ice-bath and triethylamine (1.3 mL, 9.32 mmol) was added, followed by addition of acetic anhydride (0.4 mL, 4.24 mmol) and a catalytic amount of recrystallized DMAP. The reaction was left stirring overnight and the formation of the products was confirmed by TLC. The solution was quenched with a saturated solution of NH_4_Cl (10 mL) and extracted with DCM (3 × 10 mL). The organic layers were combined and dried over MgSO_4_ and adsorbed on 10% deactivated silica gel. Products were purified through flash column chromatography (DCM:MeOH 150:1 to 100:1). Monoacetate **14** (100 mg, 0.25 mmol, 19% yield) was obtained as a white solid, diacetate **9** (246 mg, 0.57 mmol, 43% yield) was collected as a pale-yellow foam solid and the triacetate **15** (10 mg, 0.02 mmol, 1% yield) was obtained as an oil.

**9**: R*_f_* = 0.5 (DCM:MeOH 30:1); m.p. 125–128 °C (from hexane/methanol), lit. [12] 130-132 °C; $\alpha_{D}^{21}=87.10\;\left(c=1.1,{\mathrm{CHCl}}_{3}\right),\;$lit. [7] $\alpha_{D}^{24}=60\;\left(c=0.91,\mathrm{MeOH}\right),$ IR (neat) ν_max_/cm^−1^: 1771 and 1741 (s, -CO-O-), 1666 (s, -CO-N- lactam); ^1^H-NMR (300 MHz, CDCl_3_) δ 6.89 (s, 1H), 6.17 (s, 1H), 6.06 (t, J = 2.6 Hz, 1H), 6.00 (q, J = 1.3 Hz, 2H), 5.31 (dt, J = 5.4, 2.5 Hz, 1H), 4.24 (dd, J = 7.5, 5.6 Hz, 1H), 4.15–3.99 (m, 1H), 2.31 (s, 3H), 2.13 (s, 3H), 1.45 (s, 3H), 1.32 (s, 3H); ^13^C-NMR (75 MHz, CDCl_3_) δ 170.23, 169.02, 160.80, 152.30, 141.31, 133.95, 129.48, 128.58, 121.88, 113.40, 111.28, 103.03, 100.02, 78.64, 75.22, 73.77, 55.04, 27.08, 24.92, 21.05, 20.84; LRMS (EI) *m/z* 431.22 (M^+^, 23%), 389.17 (96), 331.11 (91), 289.09 (65), 169.21 (55), 155.20 (70), 141.19 (69), 127.16 (80), 113.15 (84), 99.14 (87), 85.14 (100), 71.15 (90), 57.11 (64); HRMS (EI^+^) calculated for C_21_H_21_O_9_N: 431.1211. Found 431.1209. The spectral data were matched and are in agreement with the literature data [27,37]. 

(3a*S*,3b*R*,12*S*,12a*R*)-6-hydroxy-2,2-dimethyl-5-oxo-3a,3b,4,5,12,12a-hexahydrobis([1,3]dioxolo)[4,5-c:4′,5′-j]phenanthridin-12-yl acetate (**14**) [37].

**14**: R*_f_* = 0.7 (DCM:MeOH 30:1); $\alpha_{D}^{21}=84.29\;\left(c=0.75,{\mathrm{CHCl}}_{3}\right),\;$lit. [12]$\;\alpha_{D}^{20}=85\;\left(c=0.6,{\;\mathrm{CHCl}}_{3}\right);$ m.p. 244 °C (from hexane/methanol) (decomposition), lit. [12] 248 °C; IR (neat) ν_max_/cm^−1^: 3078 (br, -OH), 2983 (w, -CH3), 2916 (w, -OCH3), 1745 (s, -CO-O-), 1676 (s, -CO-N- lactam); ^1^H-NMR (300 MHz, CDCl_3_) δ 12.87 (s, 1H), 6.66 (s, 1H), 6.50 (s, 1H), 6.12 (t, J = 2.5 Hz, 1H), 6.05 (q, J = 1.4 Hz, 2H), 5.38 (dt, J = 5.5, 2.5 Hz, 1H), 4.32 (dd, J = 7.5, 5.6 Hz, 1H), 4.23–4.06 (m, 2H), 2.21 (s, 3H), 1.54 (s, 3H), 1.40 (s, 3H); ^13^C-NMR (75 MHz, CDCl_3_) δ 170.47, 167.73, 153.21, 146.27, 134.99, 128.24, 128.12, 121.74, 111.83, 105.19, 102.62, 94.70, 78.66, 75.49, 74.17, 55.43, 27.29, 25.09, 21.29; LRMS (EI) *m/z* 389.19 (M^+^, 85%), 331.13 (100), 289.08 (89), 271.07 (54), 155.17 (53), 141.15 (58), 127.12 (60), 113.10 (63), 99.10 (62), 85.90 (68), 71.07 (54); HRMS (EI) calculated for C_19_H_19_O_8_N: 389.1105. Found 389.1108. The spectral data were matched and are in agreement with the literature data [37].

(3a*S*,3b*R*,12*S*,12a*R*)-4-acetyl-2,2-dimethyl-5-oxo-3a,3b,4,5,12,12a-hexahydrobis([1,3]dioxolo)[4,5-c:4′,5′-j]phenanthridine-6,12-diyl diacetate (**15**).

**15**: R*_f_* = 0.7 (DCM:MeOH 30:1); $\alpha_{D}^{21}=247.65\;\left(c=0.15,\;\mathrm{MeOH}\right)$; IR (neat) ν_max_/cm^−1^: 2989 (w, -CH3), 2922 (w, -OCH3), 1776 and 1743 (s, -CO-O-), 1691 (s, -CO-N- lactam); ^1^H-NMR (300 MHz, CDCl_3_) δ 6.93 (s, 1H), 6.10 (q, J = 1.2 Hz, 2H), 6.06 (t, J = 2.9 Hz, 1H), 5.58–5.50 (m, 1H), 5.47 (ddd, J = 8.6, 2.7, 1.6 Hz, 1H), 4.24 (dd, J = 7.9, 6.3 Hz, 1H), 3.91 (t, J = 8.2 Hz, 1H), 2.45 (s, 3H), 2.40 (s, 3H), 2.20 (s, 4H), 1.58 (s, 3H), 1.33 (s, 3H); ^13^C-NMR (75 MHz, CDCl_3_) δ 170.87, 170.52, 169.13, 163.66, 153.77, 141.45, 133.57, 131.42, 130.28, 125.41, 115.74, 112.74, 103.45, 100.22, 79.90, 76.58, 74.72, 53.75, 26.97, 25.83, 25.45, 21.26, 20.96; LRMS (EI) *m/z* 473.25 (M^+^, 5%), 441.38 (23), 373.13 (29), 169.21 (47) 155.20 (61), 153.18 (100), 141.19 (58), 127.16 (68), 99.14 (69), 85.14 (68), 71.15 (55); HRMS (EI^+^) calculated for C_23_H_23_O_10_N: 473.1316. Found 473.1318. 

(3a*S*,3b*S*,10b*S*,11a*R*,12*R*,12a*S*)-2,2-dimethyl-5-oxo-3a,3b,4,11a,12,12a-hexahydro-5H-bis([1,3]dioxolo)[4,5-c:4′,5′-j]oxireno[2,3-n]phenanthridine-6,12-diyl diacetate (**10**) [27].

Diacetate **9** (250 mg, 0.58 mmol) was solubilized in DCM (15 mL) and an equal volume of phosphate buffer (pH = 8.0) was added to the solution. The biphasic system was cooled to 0 °C, recrystallized *m*-CPBA (350 mg, 2.02 mmol) was added, and the reaction was left stirring overnight. The reaction was quenched with a saturated solution of Na_2_S_2_O_3_ (15 mL), transferred to a separatory funnel and further washed with NaHCO_3_ and extracted with DCM (3 × 15 mL). The combined organic layers were dried over MgSO_4_, concentrated, and adsorbed on 10% deactivated silica gel. The product was purified through flash column chromatography (*n*-hex:EA 1:1). Title compound was obtained (130 mg, 0.29 mmol, 50% yield) as a white solid. 

**10**: R*_f_* = 0.3 (n-hex:EA 1:1); m.p. 212–214 °C (from acetone), lit. [7] 224–225 °C; $\alpha_{D}^{21}=109.6\;\left(c=1.0,{\mathrm{CHCl}}_{3}\right),$ lit. [7] $\alpha_{D}^{24}=138\;\left(c=1.06,\mathrm{DCM}\right),$ ^1^H-NMR (300 MHz, CDCl_3_) δ 6.46 (s, 1H), 6.10 (dd, J = 4.9, 2.3 Hz, 2H), 5.90 (s, 1H), 5.36 (d, J = 6.1 Hz, 1H), 4.41 (dd, J = 7.9, 6.1 Hz, 1H), 4.30 (t, J = 8.1 Hz, 1H), 4.10–3.95 (m, 2H), 2.38 (s, 3H), 2.23 (s, 3H), 1.46 (s, 3H), 1.35 (s, 3H); ^13^C-NMR (75 MHz, CDCl_3_) δ 170.76, 169.06, 161.57, 152.68, 128.63, 118.83, 110.05, 103.43, 102.26, 75.93, 75.66, 74.27, 58.37, 55.25, 53.65, 27.05, 24.41, 21.24, 21.00; LRMS (ESI^+^) *m/z* 448.2 [M+H]^+^, 470.2 [M + Na]^+^, 486.1 [M + K]^+^; LRMS (EI) *m/z* 447.11 (M^+^, 5%), 405.08 (base peak), 345.05 (13), 327.05 (14), 287.03 (21), 258.04 (12), 234.05 (35); HRMS (EI) calculated for C_21_H_21_O_10_N 447.1160; found 447.1164. The spectral data were matched and are in agreement with the literature data [27]. 

(3a*S*,3b*R*,12*S*,12a*R*)-12-Cyano-11-hydroxy-2,2-dimethyl-5-oxo-3a,3b,4,5,12,12a-hexahydrobis([1,3]dioxolo)[4,5-c:4′,5′-j]phenanthridin-6-yl acetate (**11**).

The epoxide **10** (25 mg, 0.05 mmol) was suspended in a 9:1 mixture of MeCN:DCM (2 mL) under argon and KCN (10 mg, 0.15 mmol) was added to the reaction mixture, followed by LiClO_4_ (18 mg, 0.17 mmol). The reaction was left stirring overnight and no progress was observed via TLC. The reaction was brought to reflux for 4 h, filtered on a celite plug and concentrated under reduced pressure. The crude product was adsorbed on 10% deactivated silica gel and purified through flash column chromatography (DCM:MeOH:Acetone 100:1:1 to 20:1:1). Product **11** was obtained as a colorless film (5 mg, 0.01 mmol, 22% yield).

**11**: R*_f_* = 0.4 (DCM/MeOH/Acetone (20:1:1)); $\alpha_{D}^{20}=-6.53\;\left(c=0.15,\mathrm{DCM}\right)$; IR (neat) *ν*_max_/cm^−1^: 3198, 3090, 2925, 2241, 1775, 1658, 1483, 1207, 1028, 873.3; ^1^H-NMR (600 MHz, Acetone) δ 10.36 (s, 1H), 7.25 (s, 1H), 6.26 (dd, J = 5.0, 0.9 Hz, 2H), 5.35 (d, J = 4.5 Hz, 1H), 5.26 (d, J = 6.4 Hz, 1H), 4.49 (d, J = 5.4 Hz, 2H), 4.48 (d, J = 8.5 Hz, 2H), 4.09–4.03 (m, 1H), 2.31 (s, 3H), 1.45 (s, 3H), 1.42 (s, 3H); ^13^C-NMR (151 MHz, Acetone) δ 169.07, 160.03, 154.42, 140.96, 134.53, 134.27, 134.08, 118.48, 115.91, 111.30, 105.01, 104.51, 100.08, 77.81, 72.89, 69.33, 33.69, 28.28, 26.00, 20.92; LRMS (EI) *m/z* (%) 414.06 (M^+^, 10), 372.03 (80), 314.02 (20), 194.11 (20), 149.09 (39), 121.08 (57), 85.36 (54), 71.45 (84), 57.64 (100); HRMS (EI) calculated for C_20_H_18_N_2_O_8_ 414.1058; found 414.1058.

(3a*S*,3b*R*,12*R*,12a*S*)-11-hydroxy-2,2-dimethyl-5-oxo-3a,3b,4,5,12,12a-hexahydrobis([1,3]dioxolo)[4,5-c:4′,5′-j]phenanthridine-6,12-diyl diacetate (**12a**).

Diacetate-acetonide narciclasine **9** (287 mg, 0.66 mmol) was solubilized in a 2.3:1 mixture of THF:H2O (7 mL/3 mL) and cooled to 0 °C in an ice-salt bath, and the flask was covered with aluminum foil to protect it from light. Recrystallized NBS (142 mg, 0.80 mmol) was added in the solution and the colorless solution turned yellow. The reaction was left stirring in the dark and at 0 °C for 30 min and quenched with 1 mL of a saturated solution of Na_2_S_2_O_3_. The yellow color should fade, and the reaction mixture is concentrated on a rotary evaporator to remove THF, while the remaining solid product with the aqueous solution is diluted with DCM (10 mL) and extracted with DCM (3 × 10 mL). The combined organic layers are dried over MgSO_4_, filtered, and concentrated to yield 200 mg (0.44 mmol, 66% yield) of a mixture of C-1 enol (C-2 epimer) **12a** and C-1 enol **12b** as in a 1:2 ratio. After purification through flash column chromatography (DCM:MeOH:Acetone 200:1:1 to 50:1:1), **12a** was obtained as a white foam and **12b** as a white film. Note: these compounds epimerize in solution.

**12a**: R*_f_* = 0.2 (DCM:MeOH:Acetone 20:1:1); $\alpha_{D}^{21}=20.65\;\left(c=0.15,\;\mathrm{MeOH}\right);\;$IR (neat) ν_max_/cm^−1^: 3310 (br, -OH), 2985 (w, -CH3), 2926 (w, -OCH3), 1742 (s, -CO-O-), 1660 (s, -CO-N- lactam); ^1^H-NMR (600 MHz, Acetone) δ 10.22 (s, 1H), 7.18 (s, 1H), 6.21 (d, J = 2.4 Hz, 2H), 5.20 (d, J = 6.2 Hz, 1H), 5.11 (dd, J = 6.9, 3.3 Hz, 1H), 5.03 (dd, J = 9.7, 3.2 Hz, 1H), 4.73 (dd, J = 9.7, 6.2 Hz, 1H), 4.58 (d, J = 5.3 Hz, 1H), 2.30 (s, 3H), 2.13 (s, 3H), 1.42 (s, 3H), 1.39 (s, 3H); ^13^C-NMR (151 MHz, Acetone) δ 170.84, 169.09, 154.02, 140.52, 135.81, 133.77, 115.96, 111.34, 104.21, 100.44, 74.71, 73.85, 73.02, 65.05, 64.94, 28.10, 26.16, 21.09, 20.94; LRMS (EI) *m/z* 447.00 (M^+^, 5%), 405.99 (26), 344.96 (100) 326.96 (53), 311.92 (34), 286.92 (65), 258.91 (35). HRMS (EI) calculated for C_21_H_21_O_10_N 447.1160; found 447.1162.

(3a*S*,3b*R*,12*S*,12a*S*)-11-hydroxy-2,2-dimethyl-5-oxo-3a,3b,4,5,12,12a-hexahydrobis([1,3]dioxolo)[4,5-c:4′,5′-j]phenanthridine-6,12-diyl diacetate (**12b**).

**12b**: R*_f_* = 0.3 (DCM:MeOH:Acetone 20:1:1); $\alpha_{D}^{21}=11.45\;\left(c=0.35,\;\mathrm{MeOH}\right);\;$m.p. decomposes to a yellow oil at 179–181 °C (from DCM). IR (neat) ν_max_/cm^−1^: 3275 (br, -OH), 2985 (w, -CH3), 2937 (w, -OCH3), 1734 (s, -CO-O-), 1663 (s, -CO-N- lactam); ^1^H-NMR (600 MHz, DMSO) δ 11.44 (s, 1H), 7.08 (s, 1H), 6.24 (s, 1H), 6.21 (d, J = 4.7 Hz, 2H), 5.48 (d, J = 6.3 Hz, 1H), 5.09 (d, J = 6.4 Hz, 1H), 4.35 (dd, J = 9.3, 6.4 Hz, 1H), 3.78 (ddd, J = 9.6, 6.3, 3.5 Hz, 1H), 2.30 (s, 3H), 2.01 (s, 3H), 1.43 (s, 3H), 1.40 (s, 3H); ^13^C-NMR (150 MHz, DMSO) δ 170.58, 168.51, 159.32, 152.77, 139.21, 134.49, 133.94, 132.03, 114.18, 109.63, 107.13, 103.40, 98.80, 75.21, 71.56, 69.22, 68.22, 39.52, 27.93, 25.87, 20.86, 20.73; LRMS (EI) *m/z* 447.15 (M^+^, 15%), 405.15 (72), 345.11 (100), 287.09 (95), 174.14 (54), 149.09 (31), 60.55 (18); HRMS (EI) calculated for C_21_H_21_O_10_N 447.1160; found 447.1163.

(2*S*,3*R*,4*S*,4a*R*)-6-oxo-2,3,4,4a,5,6-hexahydro-[1,3]dioxolo[4,5-j]phenanthridine-2,3,4,7-tetrayl tetraacetate (**16**) [38,39].

Narciclasine **1** (100 mg, 0.33 mmol) was dissolved in DMF (3 mL) in an RBF under argon atmosphere. NEt_3_ (0.5 mL), acetic anhydride (1 mL, 1 mmol) and a few crystals of DMAP were then added immediately to the solution, which was left stirring for 4 h. The reaction mixture was rotary evaporated, and the resulting mixture was then purified using a silica gel column (1:1 Hexanes:EtOAc). This resulted in a white powder as the product **16** (102 mg, 0.21 mmol, 64%).

**16**: R*_f_* = 0.5 (20:1 DCM:MeOH); m.p.: 235 °C, lit. [38] 229–231 °C. $\alpha_{D}^{21}=218.5\;\left(c=1.67,{\;\mathrm{CHCl}}_{3}\right);\;$lit. [39] $\alpha_{D}^{24}=229\;\left(c=0.33,{\;\mathrm{CHCl}}_{3}\right);\;$IR (neat) ν_max_/cm^−1^: 3380, 3294, 2921, 2231. 1742, 1731, 1695, 1666, 1505, 1481; ^1^H-NMR (400 MHz, CDCl_3_) δ 6.88 (s, 1H), 6.38 (s, 1H), 6.10 (s, 1H), 6.03 (d, J = 3.9 Hz, 2H), 5.37 (s, 1H), 5.29 (s, 1H), 5.16 (dd, J = 9.0, 2.0 Hz, 1H), 4.53 (d, J = 8.8 Hz, 1H), 2.29 (s, 3H), 2.05 (s, 3H), 2.05 (s, 3H), 2.02 (s, 3H); ^13^C-NMR (101 MHz, CDCl_3_) δ 170.52, 169.67, 169.46, 168.86, 162.33, 152.29, 141.49, 134.17, 133.57, 131.56, 118.17, 114.85, 103.07, 101.90, 71.21, 68.27, 68.03, 50.09, 20.76, 20.62; HRMS (EI) calcd for C_22_H_21_O_11_N: 475.1115, Found: 475.1113. The spectral data were matched and are in agreement with the literature data [38,39].

(2*R*,3*S*,4*S*,4a*R*)-1-hydroxy-6-oxo-2,3,4,4a,5,6-hexahydro-[1,3]dioxolo[4,5-j]phenanthridine-2,3,4,7-tetrayl tetraacetate (**17a**).

Narciclasine tetraacetate **16** (50 mg, 0.1 mmol) was dissolved in a mixture of 3:1 THF:H_2_O (3 mL THF, 1 mL water) in an RBF under argon atmosphere. The reaction mixture was then cooled down to 0 °C using an ice bath. When at 0 °C, NBA (35 mg, 0.25 mmol) was added in one portion. The reaction mixture then turned yellow and was left to stir for 5 min. The reaction mixture was worked up by the addition of H_2_O, followed by liquid–liquid extraction. The aqueous layer was extracted with 3 × 10 mL DCM. The combined organics were then extracted with 10 mL of brine and dried over Na_2_SO_4_. The crude mixture was purified using flash column chromatography (1:1 Hexanes:EA). The resulting product was a beige powder with a 4:1 ratio (**17a**:**17b**) (35 mg, 0.07 mmol, 70%). 

**17a**: R*_f_* = 0.4 (20:1 DCM:MeOH); $\alpha_{D}^{21}=118.98\;\left(c=0.50,\;\mathrm{DCM}\right);\;$m.p.: 145–148 °C (rec. from DCM/MeOH). IR (neat) ν_max_/cm^−1^: 3311, 2986, 2919, 1735, 1655, 1480. ^1^H-NMR (300 MHz, Acetone) δ 10.24 (s, 1H), 7.11 (s, 1H), 6.53 (t, J = 5.3 Hz, 1H), 6.21 (t, J = 6.6 Hz, 2H), 6.05 (t, J = 4.1 Hz, 1H), 5.38 (dt, J = 7.9, 4.0 Hz, 1H), 4.71 (d, J = 5.4 Hz, 1H), 4.41 (dt, J = 11.1, 4.6 Hz, 1H), 3.77 (d, J = 11.1 Hz, 1H), 2.34–2.24 (m, 3H), 2.08 (s, 3H), 2.03 (s, 3H), 1.98 (s, 3H). ^13^C-NMR (75 MHz, Acetone) δ 171.96, 171.33, 171.13, 169.65, 160.81, 154.78, 141.54, 135.70, 135.03, 134.37, 116.63, 110.22, 104.93, 100.56, 69.38, 68.79, 68.32, 66.67, 31.12, 30.87, 30.61, 30.35, 30.10, 29.84, 29.84, 29.58, 21.54, 21.41, 21.31, 21.25. HRMS (EI) calcd for C_22_H_21_O_12_N: 491.1064, Found: 491.1062. 

(3a*S*,3b*R*,12*R*,12a*S*)-2,2-dimethyl-5-oxo-3a,3b,4,5,12,12a-hexahydrobis([1,3]dioxolo)[4,5-c:4′,5′-j]phenanthridine-6,11,12-triyl triacetate (**24**).

A dry solution of a mixture of C-1 enol **12a** and its C-2 epimer **12b** (75 mg, 0.16 mmol) was transferred to an rbf, solubilized in dry DCM (2 mL) and cooled to 0 °C. Triethylamine (50 µL, 0.32 mmol) was added, followed by acetic anhydride (30 µL, 0.32 mmol) and a catalytic amount of recrystallized DMAP. The reaction mixture was left stirring in the ice bath for one hour, and the TLC showed full consumption of starting material. The reaction mixture was concentrated and purified by flash column chromatography with 10% deactivated silica gel (DCM:MeOH 120:1→50:1:1). Finally, 55 mg of the title compound (0.11 mmol) was obtained as a white solid (68%).

**24**: m.p. 171–173 °C (from hexane/ethyl acetate); $\alpha_{D}^{21}=+88.10\;\left(c=0.50,\;\mathrm{MeOH}\right);\;$IR (neat) ν_max_/cm^−1^: 3196 (w, -OH), 2988 (w, -CH3), 1771 and 1739 (s, -CO-O-), 1657 (s, -CO-N- lactam); ^1^H-NMR (600 MHz, DMSO) δ 11.66 (s, 1H), 6.77 (s, 1H), 6.34 (d, J = 3.7 Hz, 1H), 6.26 (s, 1H), 6.22 (s, 1H), 5.19 (d, J = 6.1 Hz, 1H), 5.03 (dd, J = 9.8, 3.5 Hz, 1H), 4.61 (dd, J = 9.8, 6.1 Hz, 1H), 2.31 (s, 3H), 2.08 (s, 3H), 2.05 (s, 3H), 1.40 (d, J = 2.3 Hz, 6H); ^13^C-NMR (150 MHz, DMSO) δ 170.71, 169.86, 168.45, 159.26, 152.99, 139.52, 134.30, 133.33, 132.13, 114.32, 110.45, 105.73, 103.56, 98.18, 71.94, 71.45, 71.04, 64.77, 27.62, 25.86, 20.70, 20.52, 20.45; LRMS (EI) *m/z* 489.20 (M^+^, 9%), 447.15 (29), 327.07 (17), 194.05 (30), 153.12 (33), 98.99 (79). 83.93 (100), 55.91 (55); HRMS (EI+) calculated for C_23_H_23_O_11_N: 489.1266. Found 489.1261.

(3a*S*,3b*R*,12*R*,12a*S*)-2,2-dimethyl-5-(((trifluoromethyl)sulfonyl)oxy)-3a,3b,12,12a-tetrahydrobis([1,3]dioxolo)[4,5-c:4′,5′-j]phenanthridine-6,11,12-triyl triacetate (**25**).

*Method A:* Enol acetate **24** (30 mg, 0.06 mmol) was charged into a flame-dried Schlenk flask, solubilized in dry 1,2-DME (1 mL) and cooled to −5 °C in an ice-salt bath. Recrystallized 18-crown-6 ether (catalytic amount) and KH (30% in oil, one drop) were added at −5 °C under argon, and the reaction mixture turned yellow. A solution of TfCl (3.63 M in DCM, 50 µL, 0.18 mmol) was added to the reaction mixture, and the color changed to a bright yellow. The mixture was left stirring for 2 h in the ice–salt bath. The reaction progress was observed through TLC (DCM:MeOH 30:1), and new spots were observed but starting material was still present. The reaction mixture was diluted with DCM and filtered through a plug of celite; the organic phase was collected and concentrated in the rotavap. The crude product was purified by flash column chromatography with 10% deactivated silica gel (DCM:MeOH 200:1). Finally, 6 mg of title compound (0.03 mmol, 16% yield) was obtained as a film, and 18 mg of starting material was recovered. 

*Method B:* NBS (124 mg, 0.70 mmol) was added to a stirred solution of NAR acetonide diacetate **9** (150 mg, 0.35 mmol) in THF/HOAc (2 mL/2 mL). The reaction mixture was stirred for 20 min, and then it was carefully diluted with EtOAc/satd. NaHCO_3_ (15 mL/15 mL). When the gas evolution ceased, the layers were separated. The aqueous layer was further extracted with EtOAc (2 × 10 mL). The combined organic layers were washed with 5% aq. Na_2_S_2_O_3_ (2 × 10 mL), then brine (10 mL), dried over anhydrous MgSO_4_, filtered and concentrated to afford a crude solid residue of enol acetate that was used as is in the next step. To the crude product from the previous step dissolved in DCM (3 mL) at 0 °C was added pyridine (226 mL, 2.80 mmol) and Tf_2_O (235 mL, 1.40 mmol). The reaction mixture was stirred at this temperature for 3 h, after which it was quenched by the addition of 10% aq. citric acid solution (5 mL). The reaction mixture was diluted with DCM (20 mL) and water (5 mL). The layers were separated, and the organic layer was further washed with 10% aq. citric acid solution (2 × 5 mL) and brine (5 mL). The organic layer was dried over MgSO_4_, filtered, and concentrated to afford a residue that was chromatographed on silica gel using hexanes/EtOAc as an eluent (2:1 to 1:1) to afford the enol triflate product **25** as a yellow film (135 mg, 60.3% yield). 

**25**: R*_f_* = 0.8 (DCM:MeOH 30:1); $\alpha_{D}^{21}=15.85\;\left(c=0.20,\;\mathrm{MeOH}\right);\;$IR (neat) ν_max_/cm^−1^: 2926 (w, -CH3), 1750 (s, -CO-O-), 1170 (m, C-F); ^1^H-NMR (600 MHz, DMSO) δ 7.20 (s, 1H), 6.70 (d, J = 3.6 Hz, 1H), 6.45 (s, 1H), 6.40 (s, 1H), 5.39 (d, J = 6.1 Hz, 1H), 5.21 (dd, J = 9.8, 3.5 Hz, 1H), 4.74 (dd, J = 9.8, 6.1 Hz, 1H), 2.44 (s, 3H), 2.10 (s, 3H), 2.08 (s, 3H), 1.44 (s, 3H), 1.40 (s, 3H); ^13^C-NMR (150 MHz, DMSO) δ 170.32, 169.69, 167.99, 154.43, 142.40, 137.06, 124.60, 123.71, 111.35, 110.32, 104.94, 98.24, 74.77, 72.37, 70.73, 64.67, 27.50, 25.93, 20.54, 20.33, 20.06; ^19^F NMR (565 MHz, DMSO) δ -71.20; LRMS (EI) *m/z* 621 (M^+^, 5%), 579 (100), 419 (42), 286 (47), 251 (40), 165 (48), 151 (56); HRMS (EI) calculated for C_24_H_22_NO_13_F_3_S: 621.0758. Found 621.0761.

(3a*S*,3b*R*,12*R*,12a*S*)-2,2-dimethyl-5-vinyl-3a,3b,12,12a-tetrahydrobis([1,3]dioxolo)[4,5-c:4′,5′-j]phenanthridine-6,11,12-triyl triacetate (**26**).

To a stirred solution of triflate **25** (66 mg, 0.10 mmol) in dioxane (1 mL) potassium vinyl trifluoroborate (20 mg, 0.15 mmol), Pd(PPh_3_)_4_ (8 mg, 0.006 mmol), and triethylamine (21 µL, 0.15 mmol) were added. The reaction mixture was heated to 85 °C for 45 min, and it turned dark red in color. It was then cooled to RT and filtered through a celite plug. The celite cake was washed with DCM, and the organic filtrate was concentrated and adsorbed on 10% deactivated silica gel and purified by flash column chromatography (DCM:MeOH 200:1 to 150:1). The product was obtained as a light-yellow film (37 mg, 0.07 mmol, 69% yield).

**26**: R*_f_* = 0.3 (DCM:MeOH 30:1); $\alpha_{D}^{22}\;$= 79.61 (c = 0.65, DCM); IR (neat) ν_max_/cm^−1^: 2990, 2992, 1783, 1746, 1463, 1240, 1217, 1172, 1069; ^1^H-NMR (600 MHz, Acetone) δ 7.65 (dd, J = 16.8, 10.7 Hz, 1H), 7.19 (s, 1H), 6.77 (d, J = 3.5 Hz, 1H), 6.30 (dd, J = 13.4, 0.5 Hz, 2H), 6.20 (dd, J = 16.8, 2.4 Hz, 1H), 5.67–5.58 (m, 1H), 5.47 (d, J = 6.0 Hz, 1H), 5.27 (dd, J = 10.0, 3.5 Hz, 1H), 4.78 (dd, J = 10.0, 6.0 Hz, 1H), 2.44 (s, 3H), 2.09 (s, 3H), 2.08 (s, 3H), 1.49 (s, 3H), 1.43 (s, 3H); ^13^C-NMR (150 MHz, Acetone) δ 171.23, 170.73, 168.21, 155.14, 153.50, 145.82, 142.11, 136.94, 135.03, 128.93, 121.61, 121.36, 119.18, 111.01, 104.70, 98.28, 77.43, 73.99, 72.52, 66.47, 28.29, 26.36, 20.78, 20.71, 20.63; LRMS (EI) *m/z* 499 (M^+^, 40%), 457 (55), 298 (30), 297 (75), 193 (50), 149 (100), 122 (55), 121 (35), 82 (35), 71 (35); HRMS (EI) calcd for C_25_H_25_O_10_N 499.1473, found 499.1474.

(3a*S*,3b*R*,12*R*,12a*S*)-2,2-dimethyl-5-phenyl-3a,3b,12,12a-tetrahydrobis([1,3]dioxolo)[4,5-c:4′,5′-j]phenanthridine-6,11,12-triyl triacetate (**27**).

To a stirred solution of triflate **25** (60 mg, 0.096 mmol) in dioxane (1.0 mL), phenylboronic acid (17 mg, 0.139 mmol), Pd(PPh_3_)_4_ (8 mg, 0.006 mmol), and Net_3_ (20 µL, 0.143 mmol) were added. The reaction mixture was heated to 85 °C for 2 h. It was then cooled to RT and filtered through a plug of celite and washed with DCM. The organic filtrate was concentrated to afford a residue that was adsorbed on 10% deactivated silica gel and purified by flash column chromatography using DCM:MeOH as eluent (220:1 to 200:1) to afford the phenyl coupling as a white solid (32 mg, 60% yield).

**27**: R*_f_* = 0.3 (DCM:MeOH 100:1); $\alpha_{D}^{21}$ = 32.87 (c = 0.5, DCM); m.p. 270–272 °C (MeOH/pentane); IR (film) ν_max_/cm^−1^: 2927, 1778, 1743, 1462, 1216, 1173, 1045; ^1^H-NMR (600 MHz, Acetone) δ 7.54–7.49 (m, 3H), 7.45 (d, J = 6.6 Hz, 2H), 7.27 (s, 1H), 6.85 (d, J = 3.5 Hz, 1H), 6.30 (s, 2H), 6.28 (s, 1H), 5.47 (d, J = 6.0 Hz, 1H), 5.30 (dd, J = 10.0, 3.5 Hz, 1H), 4.80 (dd, J = 10.0, 6.0 Hz, 1H), 2.12 (s, 3H), 2.09 (d, J = 5.7 Hz, 3H), 1.45 (s, 3H), 1.44 (s, 3H), 1.42 (s, 3H); ^13^C-NMR (150 MHz, Acetone) δ 171.26, 170.75, 167.99, 159.03, 153.88, 145.33, 143.46, 141.93, 135.17, 128.75, 128.55, 121.28, 119.14, 111.05, 104.71, 98.29, 77.33, 73.96, 72.54, 66.49, 54.96, 28.25, 26.25, 20.78, 20.65, 19.28; LRMS (EI) *m/z* 549.18 (M^+^, 41%), 507.14 (50), 277.06 (35), 153.09 (100), 111.10 (65), 85.11 (54), 71.12 (45), 57.09 (40); HRMS (EI) calcd for C_29_H_27_O_10_N 549.1629, found 549.1629.

(3a*S*,3b*R*,12*R*,12a*S*)-2,2-dimethyl-5-(phenylethynyl)-3a,3b,12,12a-tetrahydrobis([1,3]dioxolo)[4,5-c:4′,5′-j]phenanthridine-6,11,12-triyl triacetate (**28**).

To a stirred solution of triflate **25** (40 mg, 0.056 mmol) in DMF (1 mL) Pd(PPh_3_)_2_Cl_2_ (1.4 mg, 0.002 mmol), phenyl acetylene (9.5 µL, 0.086 mmol) and NEt_3_ (27 µL, 0.195 mmol) were added. The reaction mixture was heated to 75 °C for 1 h. It was then cooled to RT and diluted with water (1 mL) and ether (10 mL). The layers were separated and the aqueous was further extracted with ether (2 × 10 mL). The organic layers were combined, dried over MgSO4, filtered and concentrated to afford a residue that was chromatographed on silica gel using hexanes/EtOAc as eluent (2:1 to 1:1) to afford the acetylene product as yellow solid (26 mg, 73% yield).

**28**: R*_f_* = 0.6 (1:1; hexanes/EtOAc); $\alpha_{D}^{21}$ = 29.5 (c = 1.3, DCM); m.p. 142–144 °C (toluene/pentane); IR (film) ν_max_/cm^−1^: 3583, 2921, 2209, 1775, 1743, 1460, 1371, 1238, 1216, 1173, 1063; ^1^H-NMR (600 MHz, DMSO) δ 7.72–7.67 (m, 2H), 7.52 (dd, J = 5.1, 1.9 Hz, 3H), 7.12 (s, 1H), 6.70 (d, J = 3.6 Hz, 1H), 6.39 (s, 1H), 6.34 (s, 1H), 5.49 (d, J = 6.1 Hz, 1H), 5.17 (dd, J = 10.0, 3.4 Hz, 1H), 4.74 (dd, J = 10.0, 6.1 Hz, 1H), 2.26 (s, 3H), 2.11 (s, 3H), 2.09 (s, 3H), 1.47 (s, 3H), 1.41 (s, 3H); ^13^C-NMR (151 MHz, DMSO) δ 170.37, 169.87, 168.29, 152.95, 145.28, 141.21, 138.59, 133.30, 131.63, 129.85, 129.06, 126.36, 121.24, 121.01, 120.05, 110.00, 104.30, 97.25, 92.58, 88.83, 75.38, 72.36, 71.06, 65.05, 27.63, 25.93, 20.57, 20.49, 20.37; LRMS (EI) *m/z* 573.06 (M^+^, 22%), 531.01 (20), 370.88 (22), 296.14 (35), 261.89 (35), 182.97 (43), 168.98 (44), 154.93 (84), 152.96 (86), 140.97 (55), 126.95 (71), 124.93 (77), 110.91 (100); HRMS (EI) calcd for C_31_H_27_O_10_N 573.1629, found 573.1619.

(2*R*,3*S*,4*S*,4a*R*)-6-phenyl-2,3,4,4a-tetrahydro-[1,3]dioxolo[4,5-j]phenanthridine-1,2,3,4,7-pentaol (**29**).

To a stirred solution of Suzuki product **27** (40 mg, 0.072 mmol) in MeOH (4 mL) was added K_2_CO_3_ (40 mg, 0.29 mmol). The reaction mixture was stirred at RT for 2 h, then the reaction was filtered through a cotton plug. To the filtrate was added 3M HCl (1.5 mL, 4.8 mmol). After stirring at RT for 4 h, it was concentrated and the crude product was adsorbed on 10% deactivated silica gel and chromatographed on 10% deactivated silica gel using CHCl_3_/MeOH (10:1) as eluent to afford the desired product as a yellow solid (18 mg, 60% yield over 2 steps). 

**29**: R*_f_* = 0.4 (CHCl_3_:MeOH 4:1); m.p. 183–185 °C (decomp., MeOH); $\alpha_{D}^{21}\;$= 61.64 (c = 0.3, DMF); IR (neat) ν_max_/cm^−1^: 3288, 2924, 2855, 1624, 1579, 1454, 1375, 1091, 1040; ^1^H-NMR (400 MHz, DMSO) δ 9.82 (s, 1H), 7.36 (d, J = 3.0 Hz, 5H), 7.19 (s, 1H), 6.14 (d, J = 3.9 Hz, 2H), 5.04 (dd, J = 12.8, 5.1 Hz, 3H), 4.78–4.68 (m, 1H), 4.66 (d, J = 1.2 Hz, 1H), 4.51–4.46 (m, 1H), 3.98 (s, 2H); ^13^C-NMR (100 MHz, DMSO) δ 157.33, 151.22, 146.61, 144.15, 135.47, 135.37, 133.29, 128.79, 126.68, 123.35, 116.07, 101.59, 93.25, 71.76, 68.28, 66.18; LRMS (EI) *m/z* 277.05 (33%), 262.06 (60), 119.08 (85), 118.08 (100); HRMS (EI) calcd for C_20_H_17_O_7_N 383.1000, found 383.0989.

(2*R*,3*S*,4*S*,4a*R*)-6-(phenylethynyl)-2,3,4,4a-tetrahydro-[1,3]dioxolo[4,5-j]phenanthridine-1,2,3,4,7-pentaol (**30**).

To a stirred solution of Sonogashira product **28** (10 mg, 0.019 mmol) in MeOH (1 mL) was added K_2_CO_3_ (10 mg, 0.072 mmol). The reaction mixture was stirred at RT for 30 min, then the reaction was filtered. To the filtrate was added 3M HCl (0.4 mL, 1.2 mmol). After stirring at RT for 3 h, it was directly evaporated on silica gel and chromatographed on silica gel using DCM/MeOH (3:1) as eluent to afford the desired product as a yellow solid (3 mg, 39.2% yield over two steps.) 

**30**: R*f* = 0.5 (3:1; DCM/MeOH); $\alpha_{D}^{21}\;$= −93.0 (c = 0.20, DCM/MeOH (3:1)); m.p. 178–180 °C (MeOH/Et_2_O); IR (film) ν_max_/cm^−1^: 3432, 2920, 2849, 1639, 1502, 1460, 1097, 1032, 1015; ^1^H-NMR (300 MHz, DMSO) δ 7.93 (dd, J = 6.5, 2.8 Hz, 2H), 7.54 (dd, J = 5.2, 1.5 Hz, 3H), 7.12 (s, 1H), 7.04 (s, 1H), 6.27 (s, 2H), 5.06 (d, J = 4.6 Hz, 1H), 5.01 (d, J = 5.5 Hz, 1H), 4.87 (s, 1H), 4.72 (s, 1H), 4.58 (s, 1H), 3.92 (s, 2H); ^13^C-NMR (75 MHz, DMSO) δ 155.20, 152.33, 149.39, 148.82, 134.10, 132.09, 131.37, 130.51, 130.00, 129.00, 124.93, 122.07, 115.47, 104.21, 102.79, 95.21, 71.78, 68.32, 67.98, 65.85; LRMS (EI) *m/z* 407.09 (M^+^, 10%), 355.06 (20), 277.04 (20), 262.04 (90), 182.98 (40), 68.93 (100), 50.85 (40); HRMS (EI) calcd for C_22_H_17_O_7_N, 407.1000 found 407.1000. 

(2*R*,3*S*,4*S*,4a*R*)-1,2,3,4,7-pentahydroxy-3,4,4a,5-tetrahydro-[1,3]dioxolo[4,5-j]phenanthridin-6(2H)-one (**31**).

To a solution of **17a** (52mg, 0.11 mmol) in THF (4 mL), concentrated HCl (1 mL) was added dropwise. The solution was left to stir for 16 h. Solid NaHCO_3_ was added until the gas stopped evolving, and the pH was neutral. The solution was then filtered and evaporated under reduced pressure. The crude mixture was then purified via flash column chromatography (DCM:MeOH 10:1), resulting in a white amorphous solid as the product (5 mg, 0.015 mmol, 14%). 

**31**: R*_f_* = 0.2 (CHCl3:MeOH 4:1); $\alpha_{D}^{21}=13.89\;\left(c=0.10,\mathrm{MeOH}\right),$ IR (neat) ν_max_/cm^−1^: 3301 (br, free -OH), 2927 and 2858 (s, m, -CH-), 1672 (s, -CO-N- lactam); ^1^H-NMR (400 MHz, DMSO) δ 13.55 (s, 1H), 11.59 (s, 1H), 6.85 (s, 1H), 6.13 (s, 2H), 5.11 (d, J = 5.1 Hz, 1H), 4.89 (d, J = 5.6 Hz, 1H), 4.72 (d, J = 5.2 Hz, 1H), 4.67 (d, J = 5.6 Hz, 1H), 4.46 (dd, J = 5.0, 3.4 Hz, 1H), 3.83–3.79 (m, 2H); ^13^C-NMR (100 MHz, DMSO) δ 165.82, 153.39, 143.41, 135.35, 134.81, 130.90, 112.59, 108.07, 102.06, 94.02, 67.72, 67.37, 67.19, 65.35; LRMS (ESI^−^) *m/z* 322 [M + H]^+^, 346 [M + Na]^+^.

(7a*R*,8*S*,9*S*,10*R*)-5,6,7a,8,9,10-hexahydro-[1,3]dioxolo[4,5-j]pyrano[4,3,2-gh]phenanthridine-8,9,10,11-tetraol (**33**).

To a stirred solution of **26** (37 mg, 0.075 mmol) in MeOH (3 mL) was added K_2_CO_3_ (37 mg, 0.26 mmol). The reaction mixture was stirred at RT overnight (18 h), then the reaction was filtered through a cotton plug. To the filtrate was added 3M HCl (1.5 mL, 4.8 mmol). After stirring at RT for 3 h, the reaction mixture was concentrated, and the crude product was adsorbed on 10% deactivated silica gel and purified by column chromatography on 10% deactivated silica gel using CHCl_3_:MeOH (10:1 to 6:1) as eluent to afford the product as an orange solid. The product was further recrystallized in methanol to obtain a white solid (15 mg, 0.045 mmol, 60% yield over 2 steps).

**33**: R*_f_* = 0.3 (CHCl_3_:MeOH 4:1), $\alpha_{D}^{22}=-1.63\;\left(c=0.13,\;\mathrm{DMSO}\right);\;$ m.p. 230 °C (decomp, rec. from MeOH); IR (neat) ν_max_/cm^−1^: 3350, 2925, 1697, 1658, 1459, 1243, 1097, 1031; ^1^H-NMR (600 MHz, DMSO) δ 7.15 (s, 1H), 6.18 (s, 2H), 5.08 (d, J = 4.7 Hz, 1H), 5.02 (s, 1H), 4.99 (d, J = 5.5 Hz, 1H), 4.73–4.68 (m, 1H), 4.66 (s, 1H), 4.52 (dd, J = 9.1, 3.9 Hz, 2H), 4.49 (d, J = 2.7 Hz, 1H), 3.94 (s, 2H), 3.24 (dd, J = 7.1, 5.5 Hz, 2H); ^13^C-NMR (150 MHz, DMSO) δ 152.24, 152.11, 147.66, 134.93, 132.88, 131.31, 123.50, 114.13, 102.19, 94.19, 71.82, 68.24, 68.07, 67.01, 65.92, 31.76. LRMS (EI) *m/z* 333.07 (M^+^, 10%), 281.11 (10), 157.05 (15), 84.07 (100), 66.05 (47), 60.05 (26); HRMS (EI) calcd for C_16_H_15_O_7_N, 333.0843 found 333.0847.

*Cell Viability Assay:* Cell viability was measured by MTT (3-(4,5-dimethylthiazol-2-yl)-2,5-diphenyl tetrazolium bromide) assay in three cell lines: neuroblastoma cell line BE(2)-C, lung squamous cell carcinoma cell line H157 and lung adenocarcinoma cell line A549 (all obtained from American Type Culture Collection). BE(2)-C cells were maintained in Dulbecco’s Modified Eagle Medium/Nutrient Mixture F-12 (DMEM/F12) media supplemented with 10% fetal bovine serum (FBS). H157 and A549 cells were maintained in RPMI-1640 media supplemented with 10% FBS. For measuring the IC_50_ values of cell viability, 3000 cells per well were plated into a 96-well plate, and cells were treated with a series of dilutions (from 500 µM to 0.32 nM) of individual compounds in triplicate for four days. After four days, cells were replaced with MTT reagent (at 0.25 mg/mL in culture media) in each well and incubated with cells for 2 h at 37 °C. Culture media was removed after microplates were spun at 2000 rpm for 5 min. DMSO was then used to dissolve the crystals formed in each of the 96 wells. Optical density values at wavelength 570 nm and 630 nm were measured using SpectraMax 190 (Molecular Devices, San Jose, CA, USA). The difference in the two optical density values was used to analyze the relative cell survival in each well. IC_50_ values were calculated using Graphpad Prism software.

## 4. Conclusions

Throughout our attempts to synthesize C-1 derivatives of narciclasine, whose biological activity could be compared with those of the corresponding C-1 unnatural derivatives of pancratistatin, four novel C-1 and C-6 analogues were obtained. It was observed that installation of the C-1 enol moiety enhanced the reactivity of the B-ring lactam, so that triflation reactions yielded an imino triflate/triflyl imidate and subsequent cross-couplings occurred at the C-6 position. The four new analogues were subjected to biological testing and the SAR database was expanded with results that indicate that the removal of the lactamic carbonyl has a deleterious effect on activity, although it does not abolish it altogether. Our efforts will continue in order to design a new route taking such activity into consideration and to hopefully synthesize C-1 analogues of narciclasine that could be directly compared to similar pancratistatin derivatives developed and evaluated previously [8,9]. We will report any new results in due course.

## Figures and Tables

**Table 1:** Results of biological evaluation.

Compound	IC_50_, µM ^a^		
	BE(2)-C ^b^	H157 ^c^	A549 ^d^
**1**	0.036	0.033	0.061
**2**	0.285	0.173	0.527
**29**	5.42	5.37	21.9
**30**	112.4	113.0	26.6
**31**	>500	>500	>500
**33**	>500	>500	>500

^a^ Concentration required to reduce the viability of cells by 50% after 4 days of treatment with the indicated compounds relative to a DMSO control, as determined by the MTT assay. ^b^ Human neuroblastoma cell line ATCC CRL-2268. ^c^ Human lung squamous cell carcinoma ATCC CRL-5802. ^d^ Human lung adenocarcinoma cell line ATCC CCL-185.

## Data Availability

Not applicable.

## Supplementary Materials

The following supporting information can be downloaded at: https://www.mdpi.com/article/10.3390/molecules27134141/s1. Figure S1: Biological Activity; S2: ^1^H-NMR of **6**; S3. ^13^C-NMR of **6**; S4: ^1^H-NMR of **9**; S5. ^13^C-NMR of **9**; S6: ^1^H-NMR of **10**; S7. ^13^C-NMR of **10**; S8: ^1^H-NMR of **11**; S9. ^13^C-NMR of **11**; S10: ^1^H-NMR of **12a**; S11. ^13^C-NMR of **12a**; S12: ^1^H-NMR of **12b**; S13. ^13^C-NMR of **12b**; S14: ^1^H-NMR of **14**; S15. ^13^C-NMR of **14**; S16: ^1^H-NMR of **15**; S17. ^13^C-NMR of **15**; S18: ^1^H-NMR of **16**; S19. ^13^C-NMR of **16**; S20: ^1^H-NMR of **17a**; S21. ^13^C-NMR of **17a**; S22: ^1^H-NMR of **24**; S23. ^13^C-NMR of **24**; S24: ^1^H-^15^ N HSQC of **24**; S25. ^1^H-^15^N HMBC of **24**; S26: ^1^H-NMR of **25**; S27. ^13^C-NMR of **25**; S28: ^19^F-NMR of **25**; S29: ^1^H-NMR of **26**; S30. ^13^C-NMR of **26**; S31: ^1^H-^15^N HMBC of **26**; S32. ^1^H-NMR of **27**; S33. ^13^C-NMR of **27**; S34: ^1^H-NMR of **28**; S35. ^13^C-NMR of **28**; S36: ^1^H-NMR of **29**; S37. ^13^C-NMR of **29**; S38: ^1^H-NMR of **30**; S39. ^13^C-NMR of **30**; S40: ^1^H-NMR of **31**; S41. ^13^C-NMR of **31**; S42: ^1^H-NMR of **33**; S43. ^13^C-NMR of **33**.

## Sample Availability

Samples of the compounds are not available from the authors.
