# Supplementary material for: Conversion of Natural Narciclasine to Its C-1 and C-6 Derivatives and Their Antitumor Activity Evaluation: Some Unusual Chemistry of Narciclasine
Source: Molecules. 2022 Jun 28;27(13):4141. doi: 10.3390/molecules27134141 (PMC9268329; doi:10.3390/molecules27134141)

Supporting Material

Conversion of natural narciclasine to its C-1 and C-6 derivatives and their biological evaluation. Some unusual chemistry of narciclasine.

Juana Goulart Stollmaier [1,*], Jared Thomson [1], Mary Ann Endoma-Arias [1], Razvan Simionescu [1], Alexandra Vernaza [2], Nakya Mesa-Diaz [2], Mitchell Smith [2], Liqin Du [2], Alexander Kornienko [2,*], and Tomas Hudlicky[1]

[1] Department of Chemistry, Brock University, 1812 Sir Isaac Brock Way, St. Catharines, ON L2S 3A1, Canada; jt15gq@brocku.ca (J.T.); maryann.arias@bpt.eurofinsca.com (M.A.E-A.); rsimionescu@brocku.ca (R.S.) thudlicky@brocku.ca (T.H.)

[2] Department of Chemistry and Biochemistry, Texas State University, San Marcos, TX 78666, USA; a_v651@txstate.edu (A.V.); nlm59@txstate.edu (N.M-D.); mts85@txstate.edu (M.S.); l_d141@txstate.edu (L.D.)

* Correspondence: juanagstollmaier@gmail.com (J.G.S); a_k76@txstate.edu (A.K.)

Table of Contents

Figure S1. Biological Activity 4

Selected Spectra 5

Figure S2. ^{1}H-NMR of **6** 5

Figure S3. ^{13}C-NMR of **6** 5

Figure S4. ^{1}H-NMR of **9** 6

Figure S5. ^{13}C-NMR of **9** 6

Figure S6. ^{1}H-NMR of **10** 7

Figure S7. ^{13}C-NMR of **10** 7

Figure S8. ^{1}H-NMR of **11** 8

Figure S. ^{13}C-NMR of **11** 8

Figure S10. ^{1}H-NMR of **12a** 9

Figure S11. ^{13}C-NMR of **12a** 9

Figure S12. ^{1}H-NMR of **12b** 10

Figure S13. ^{13}C-NMR of **12b** 10

Figure S14. ^{1}H-NMR of **14** 11

Figure S15. ^{13}C-NMR of **14** 11

Figure S16. ^{1}H-NMR of **15** 12

Figure S17. ^{13}C-NMR of **15** 12

Figure S18. ^{1}H-NMR of **16** 13

Figure S19. ^{13}C-NMR of **16** 14

Figure S20. ^{1}H-NMR of **17a** 15

Figure S21. ^{13}C-NMR of **17a** 15

Figure S22. ^{1}H-NMR of **24** 16

Figure S23. ^{13}C-NMR of **24** 16

Figure S24. ^{1}H-^{15}N HSQC of **24** 17

Figure S25. ^{1}H-^{15}N HMBC of **24** 17

Figure S26. ^{1}H-NMR of **25** 18

Figure S27. ^{13}C-NMR of **25** 18

Figure S28. ^{19}F-NMR of **25** 19

Figure S29. ^{1}H-NMR of **26** 20

Figure S30. ^{13}C-NMR of **26** 20

Figure S31. ^{1}H-^{15}N HMBC of **26** 21

Figure S32. ^{1}H-NMR of **27** 22
Figure S33. ^{13}C-NMR of **27** 22
Figure S34. ^{1}H-NMR of **28** 23
Figure S35. ^{13}C-NMR of **28** 23
Figure S36. ^{1}H-NMR of **29** 24
Figure S37. ^{13}C-NMR of **29** 24
Figure S38. ^{1}H-NMR of **30** 25
Figure S39. ^{13}C-NMR of **30** 25
Figure S40. ^{1}H-NMR of **31** 26
Figure S41. ^{13}C-NMR of **31** 26
Figure S42. ^{1}H-NMR of **33** 27
Figure S43. ^{13}C-NMR of **33** 27

Figure S1. Biological Activity

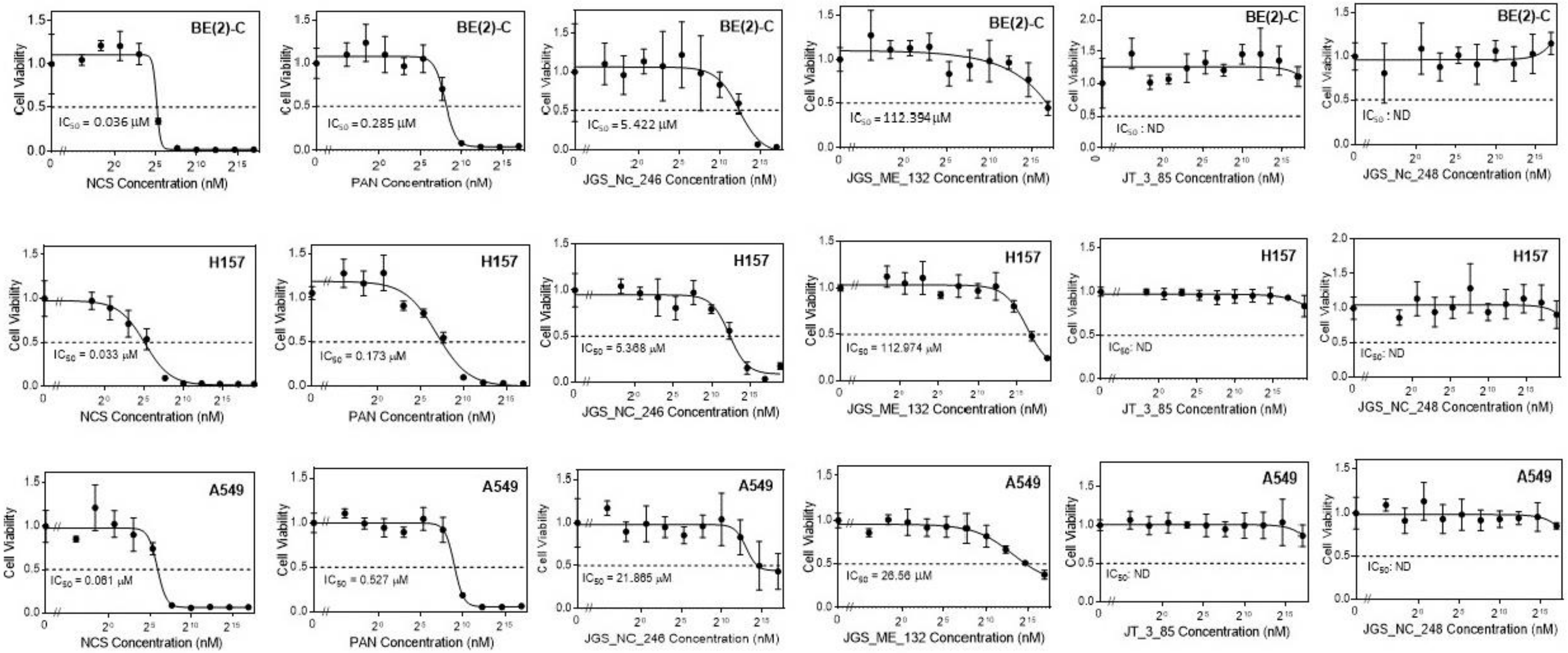

Suppl. Figure S1. Dose-dependent cytotoxicity of compounds in cancer cell lines. Cells were treated with a serial of dilutions of each compound in three replicates in 96-well plates for 4 days, and cell viability was measured using MTT assay. Shown in each figure is dose-dependent response of the indicated cells to the indicated compound, with the dotted line signifying the IC_{50}. ND, not determined (>500 µM). NCS = **1**, PAN = **2**, JGS_Nc_246 = **29**, JGS_ME_132 = **30**, JT_3_85 = **31**, JGS_Nc_248 = **33**.

Selected Spectra

Figure S2. ^{1}H-NMR of **6**

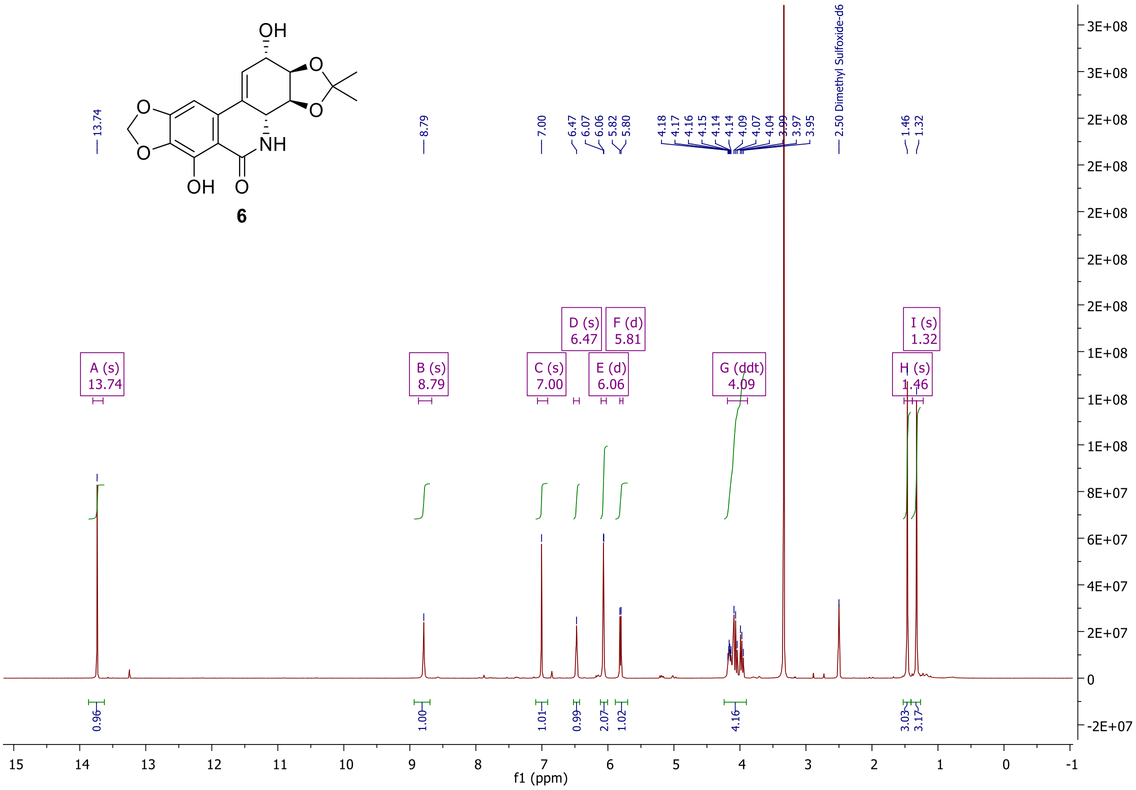

Figure S3. ^{13}C-NMR of **6**

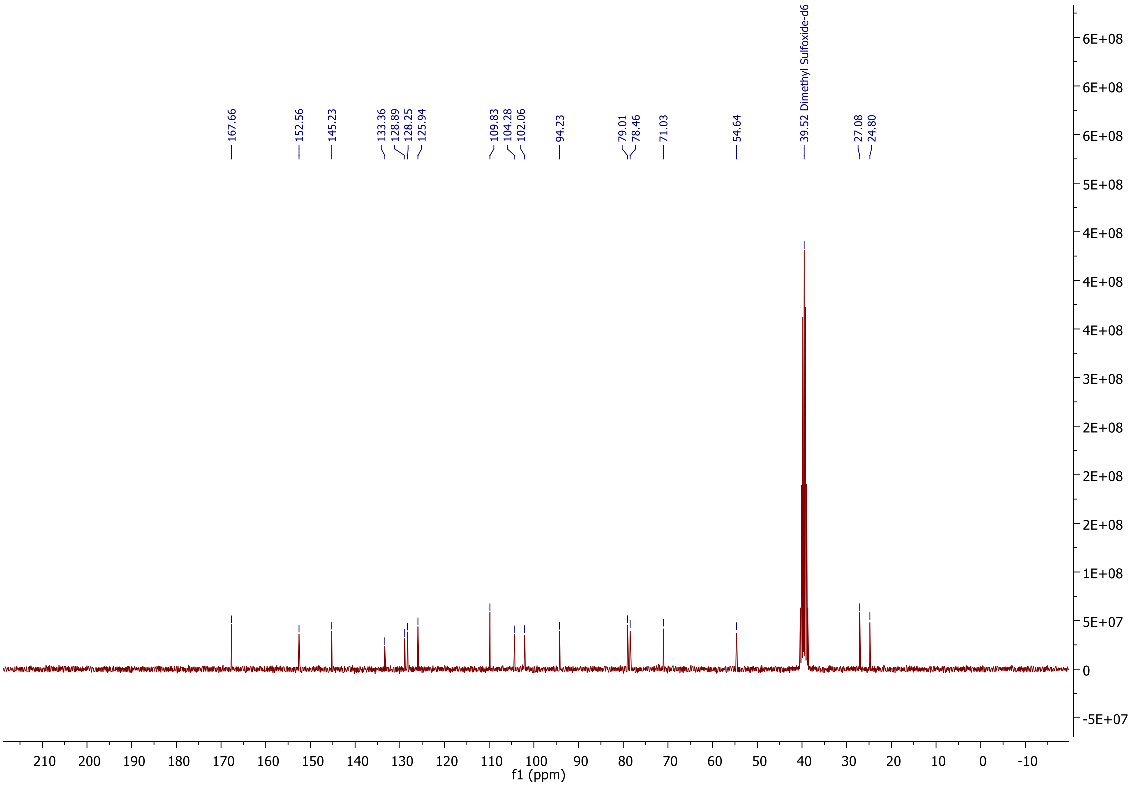

Figure S4. ¹H-NMR of **9**

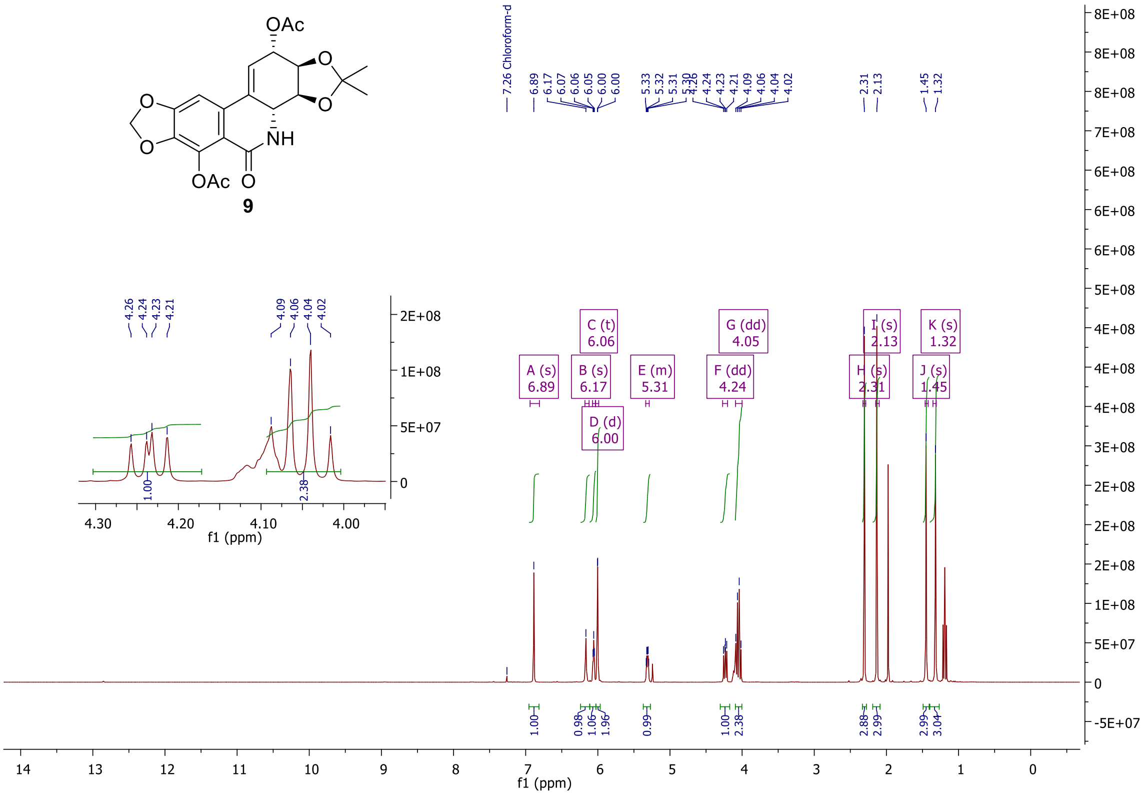

Figure S5. ¹³C-NMR of **9**

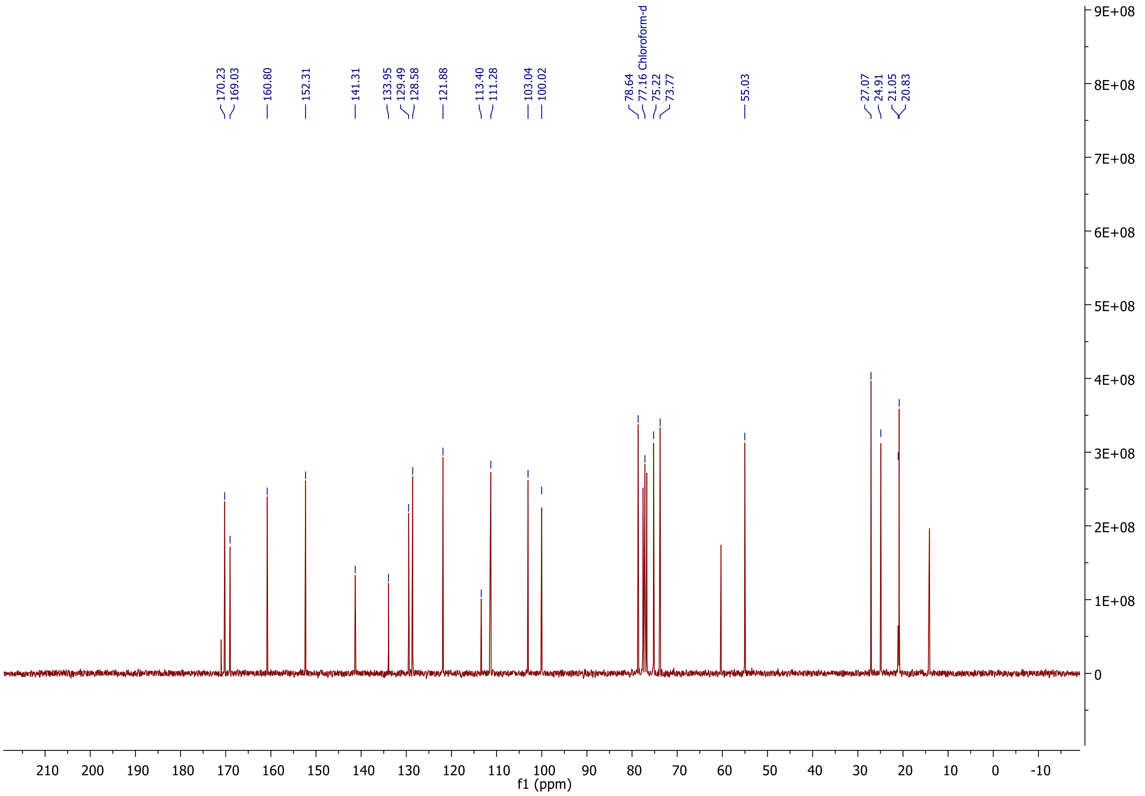

Figure S6. ^{1}H-NMR of **10**

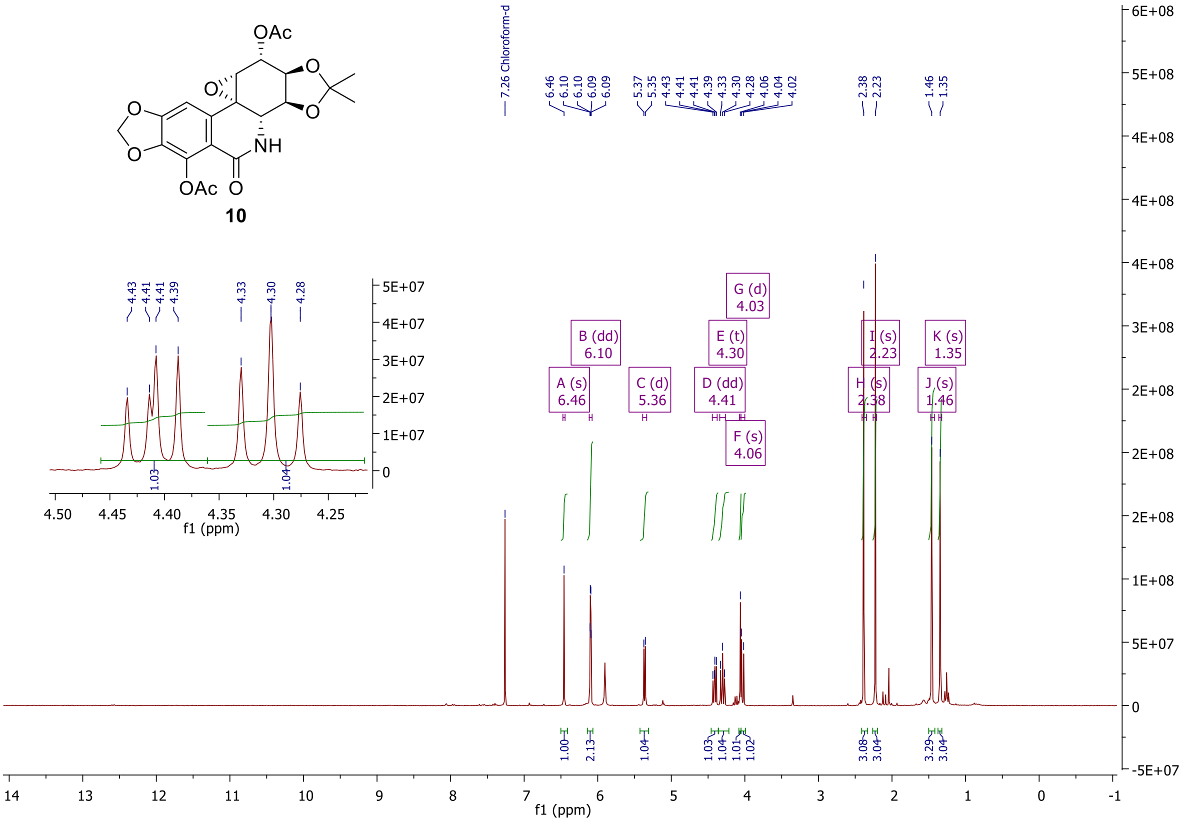

Figure S7. ^{13}C-NMR of **10**

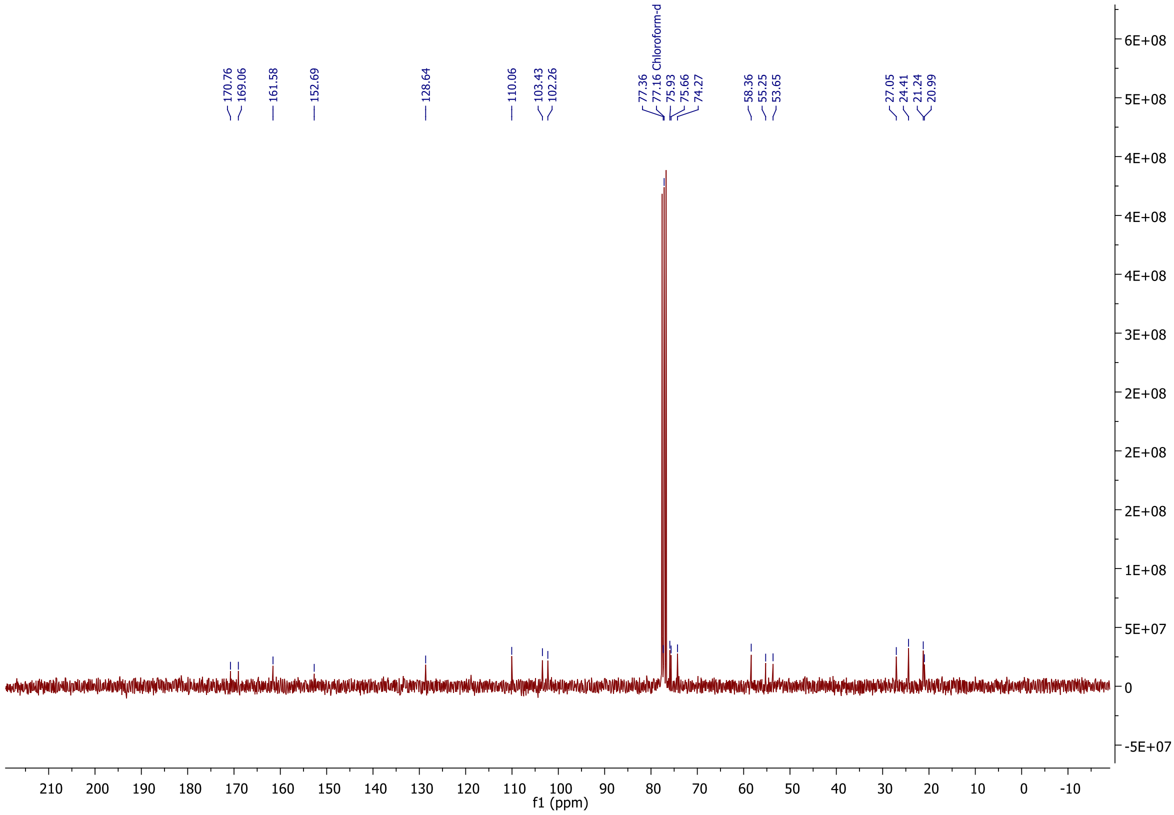

Figure S8. ^{1}H-NMR of **11**

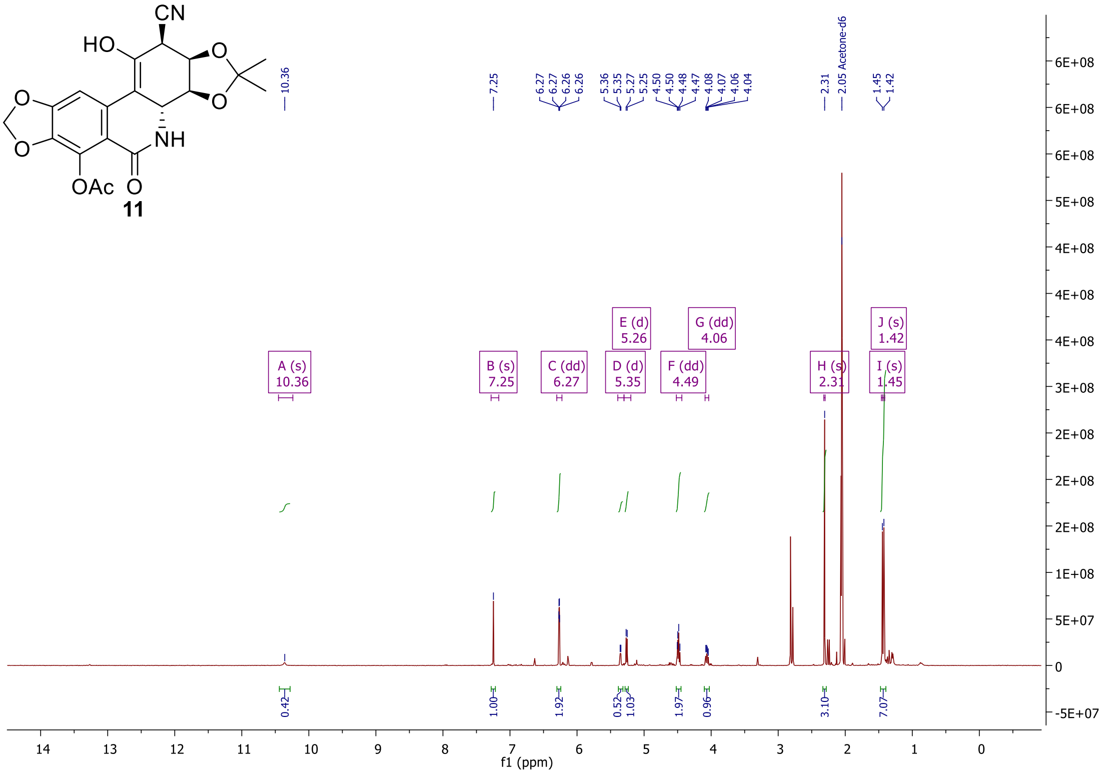

Figure S9. ^{13}C-NMR of **11**

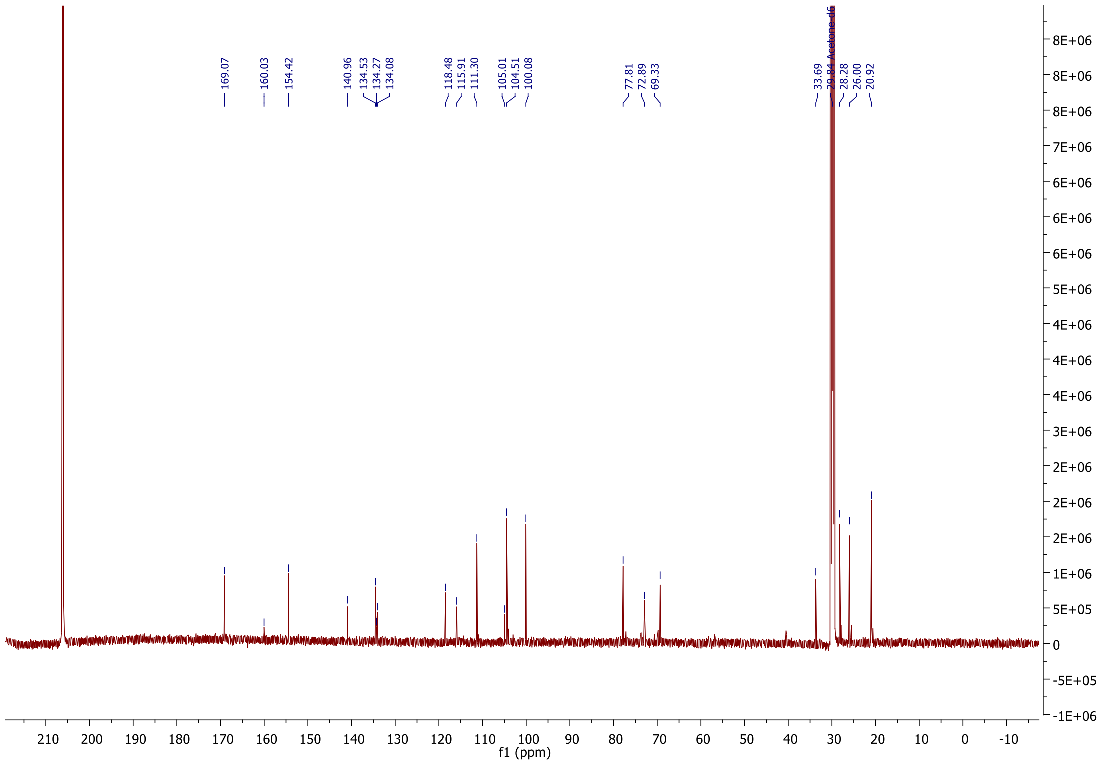

Figure S10. ^{1}H-NMR of **12a**

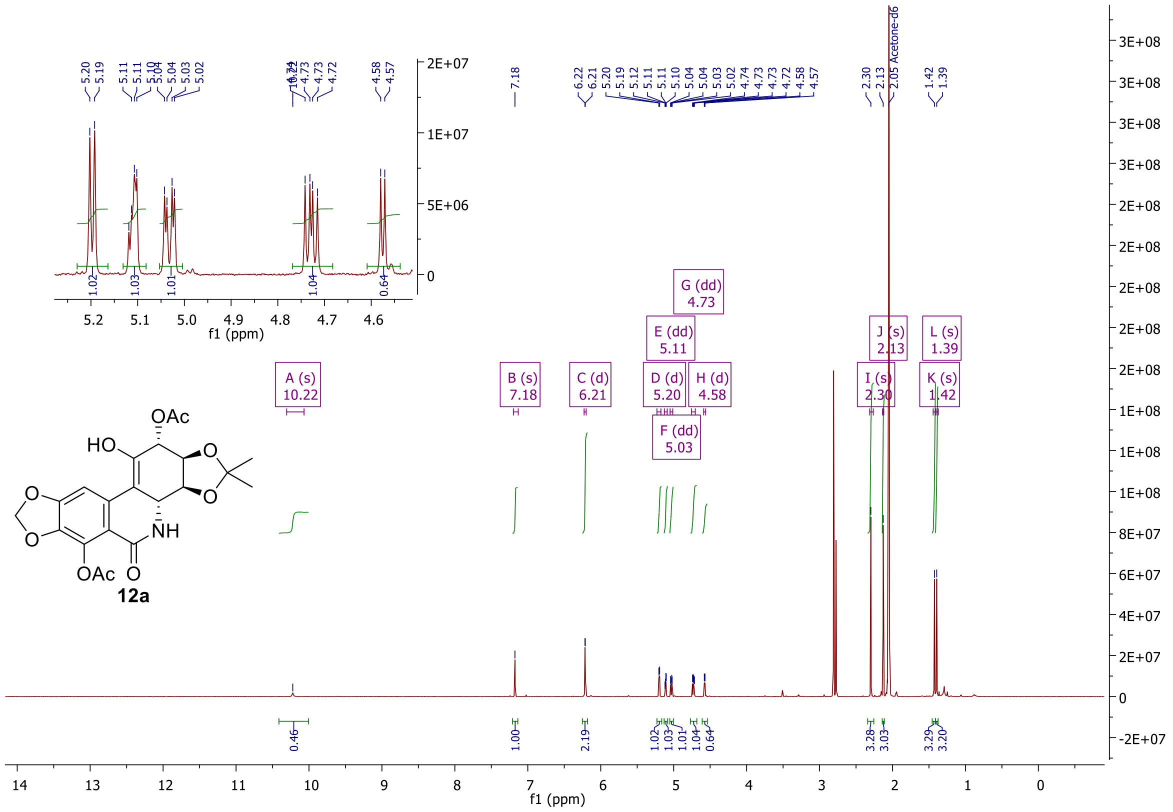

Figure S11. ^{13}C-NMR of **12a**

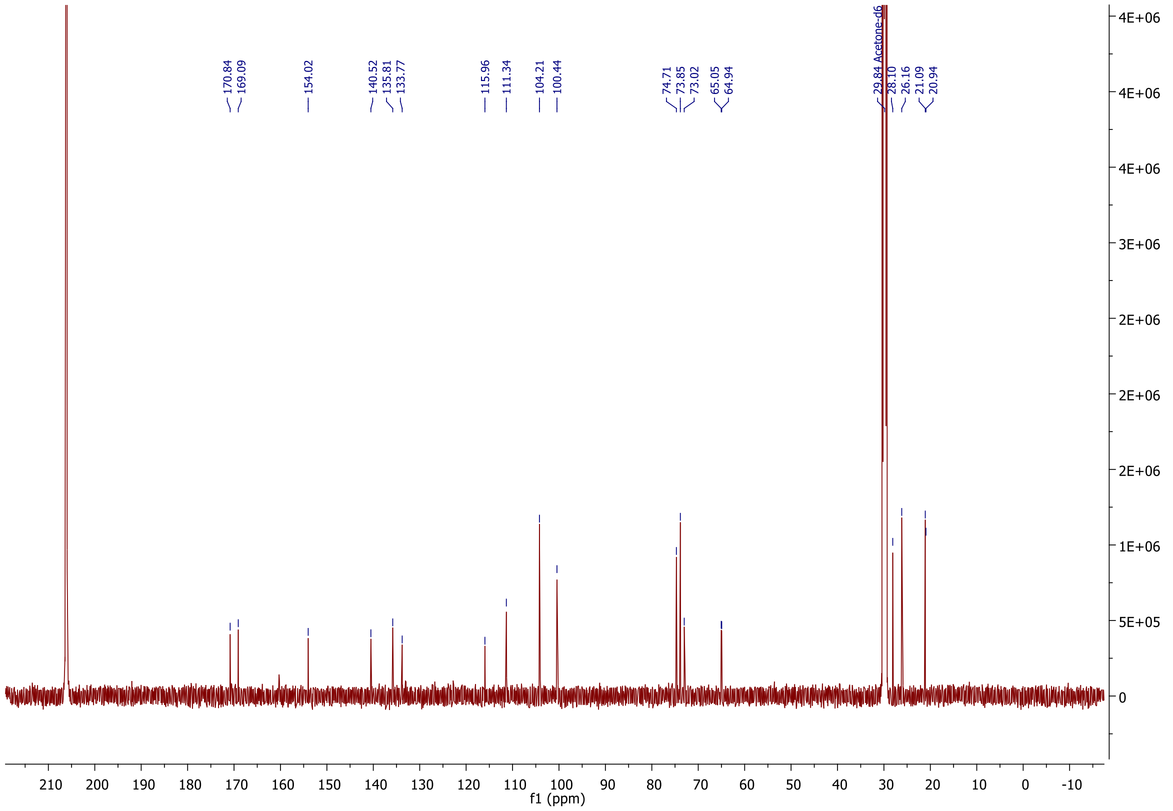

Figure S12. ^{1}H-NMR of **12b**

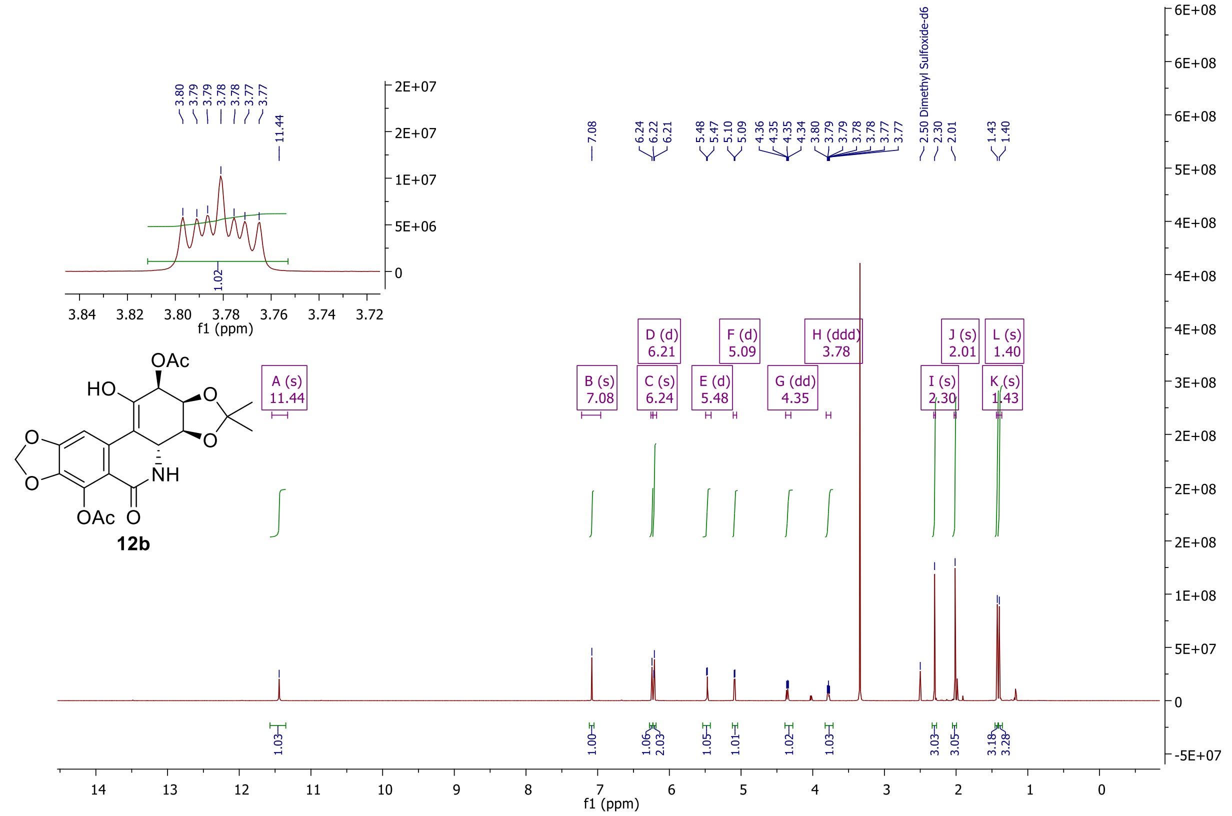

Figure S13. ^{13}C-NMR of **12b**

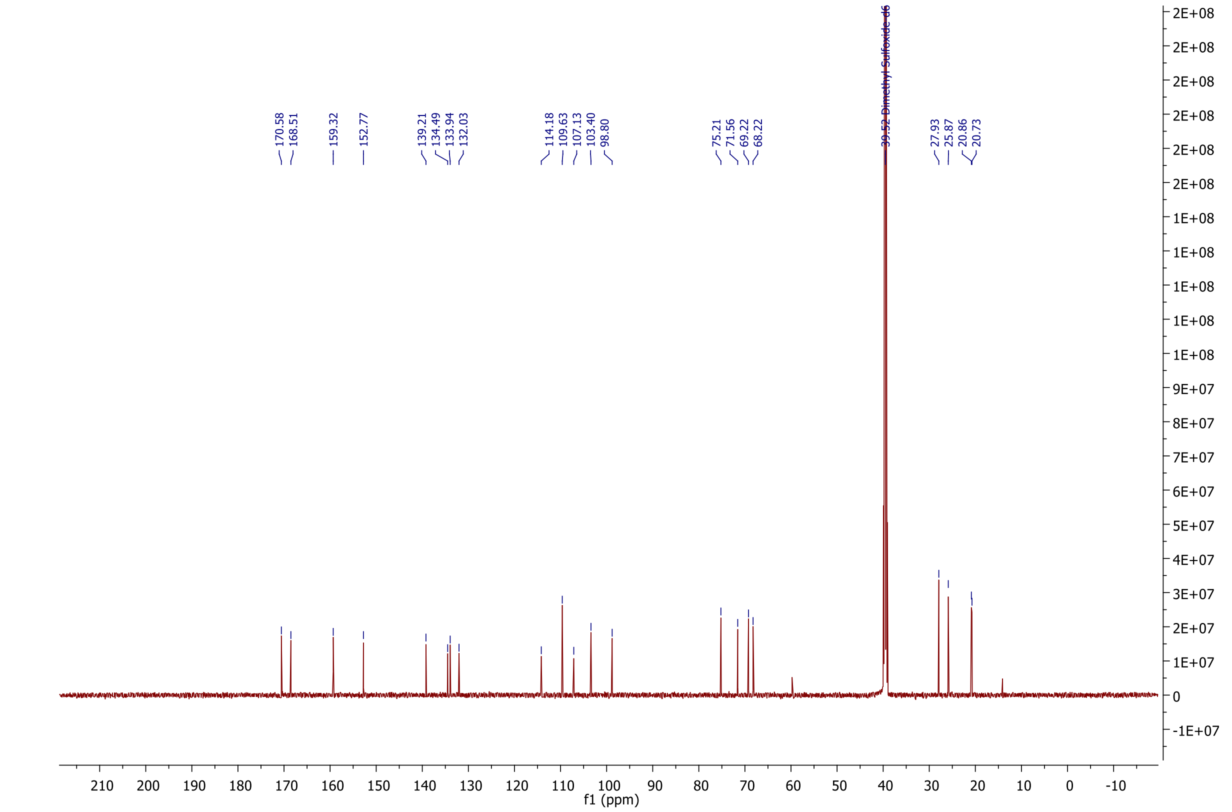

Figure S14. ^{1}H-NMR of **14**

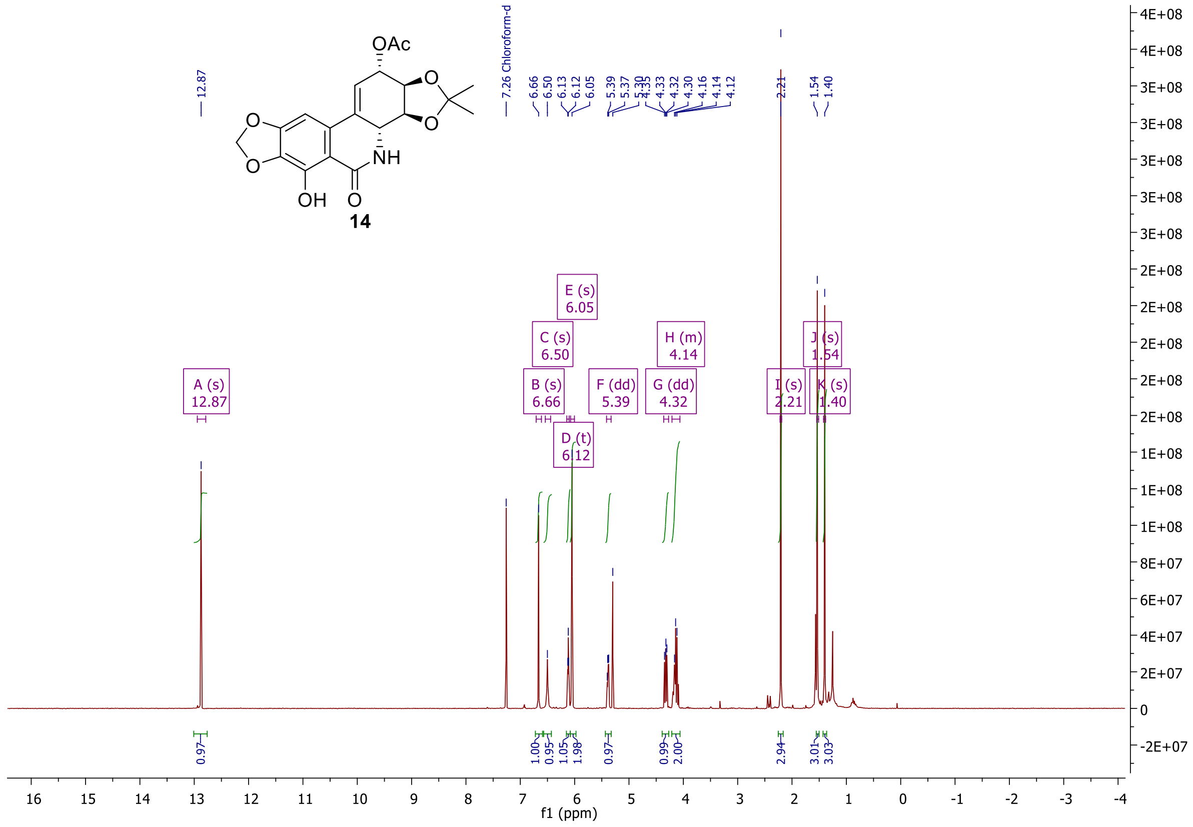

Figure S15. ^{13}C-NMR of **14**

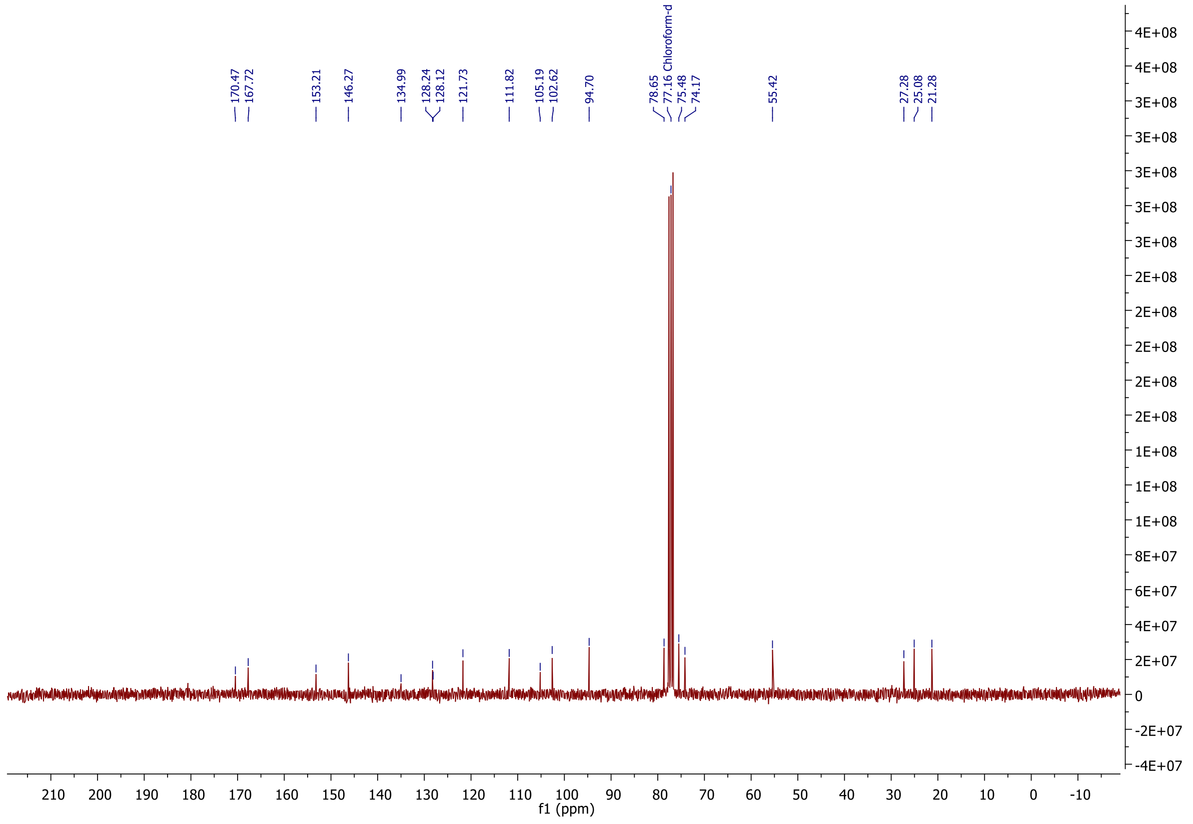

Figure S16. ^{1}H-NMR of **15**

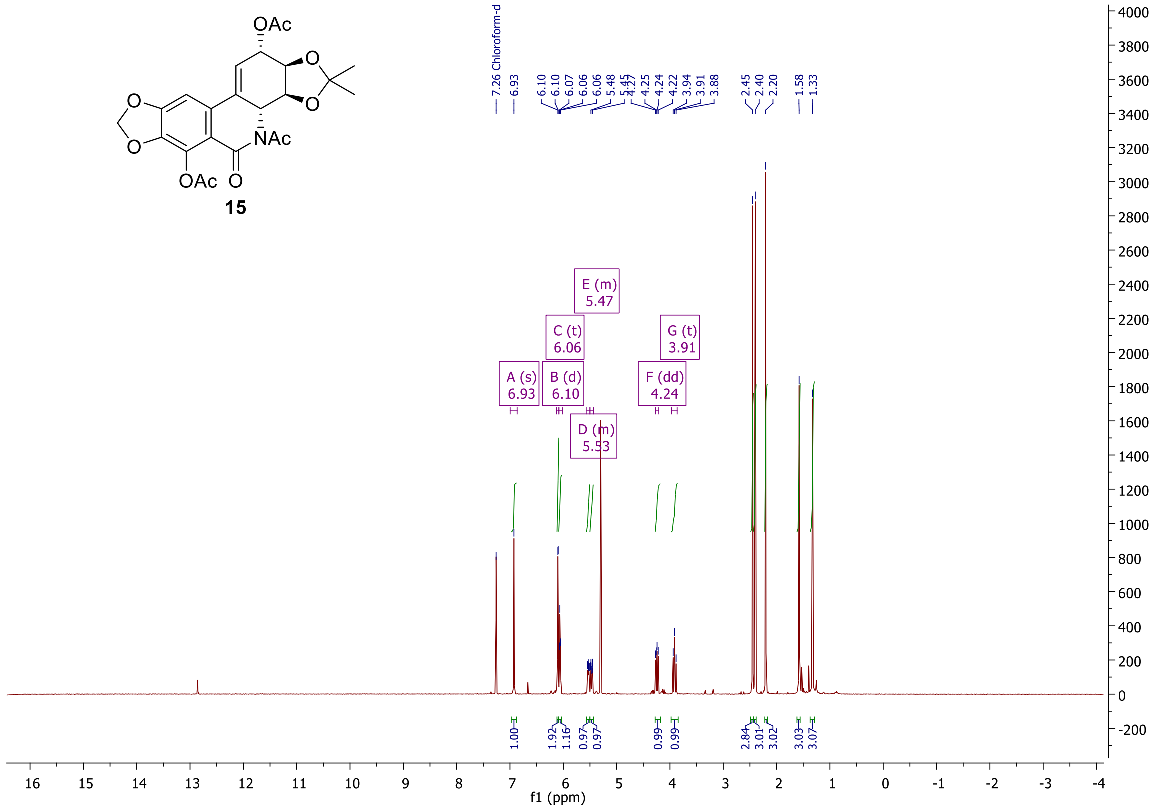

Figure S17. ^{13}C-NMR of **15**

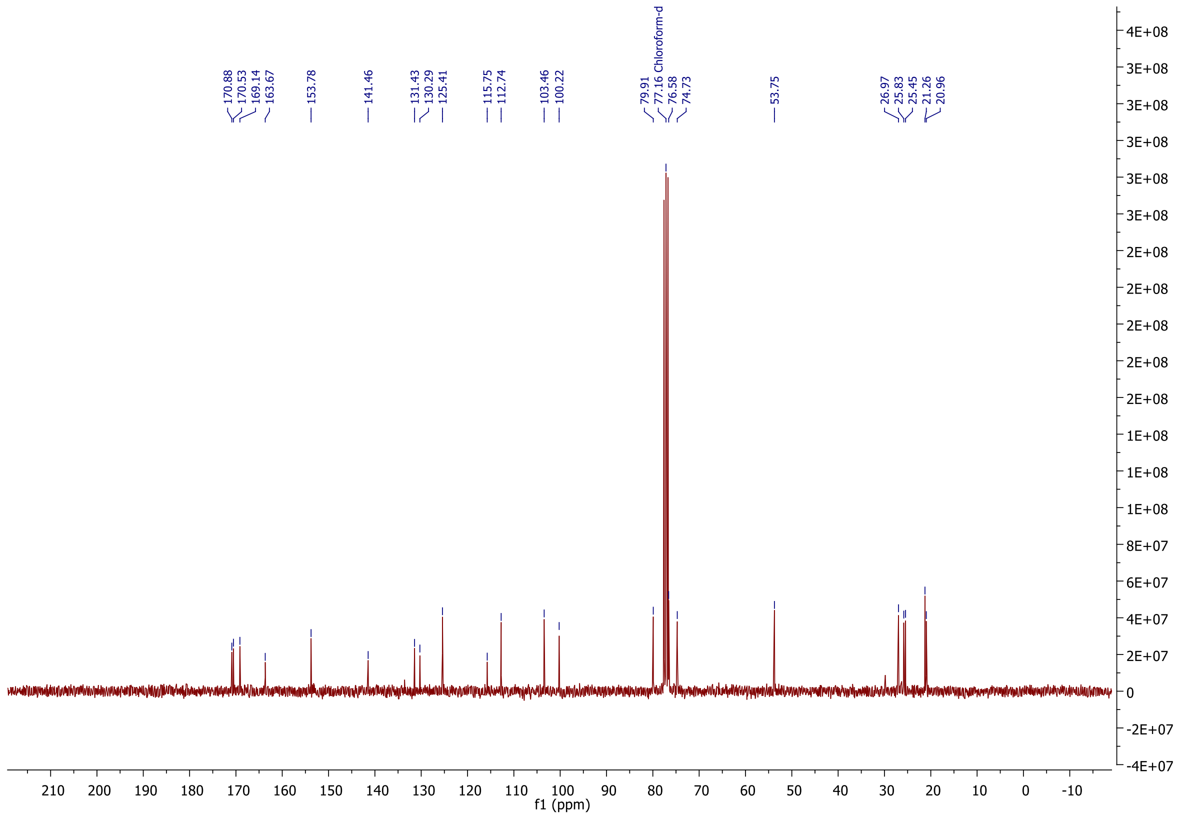

Figure S18. ^{1}H-NMR of **16**

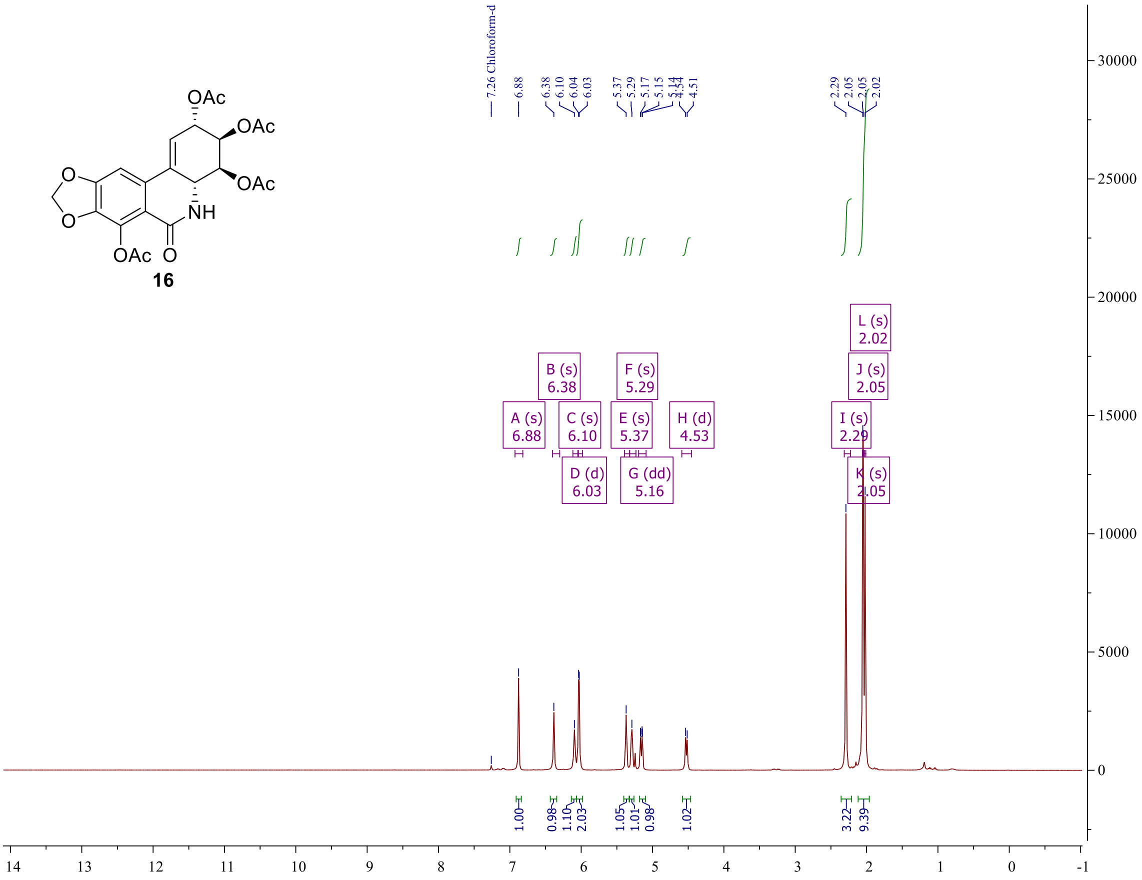

Figure S19. ^{13}C-NMR of **16**

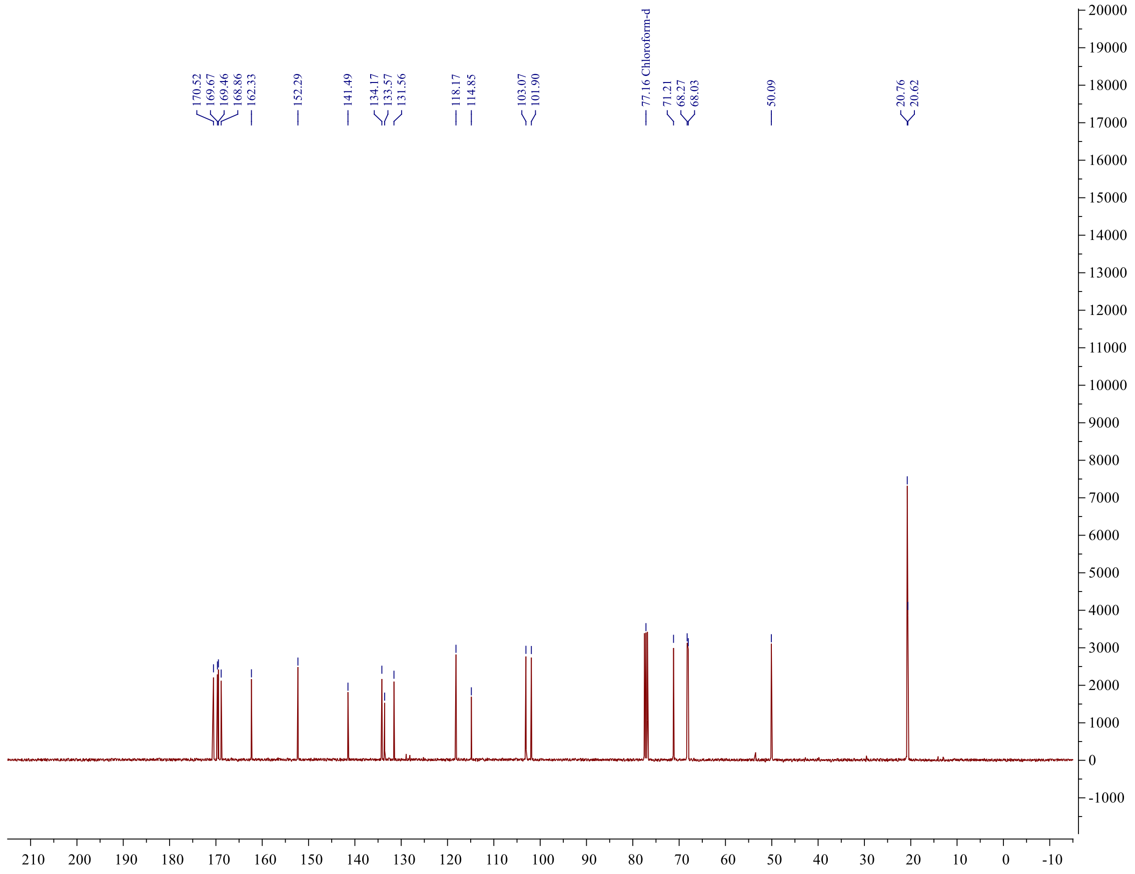

Figure S20. ^{1}H-NMR of **17a**

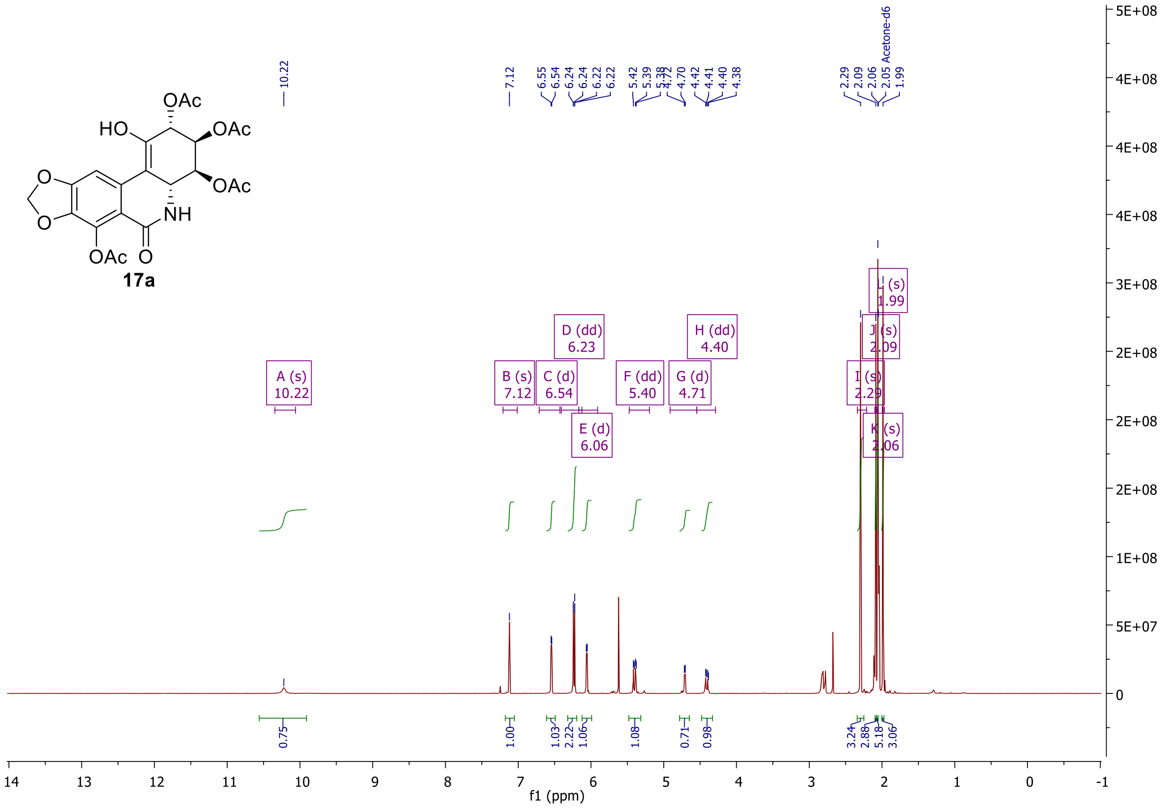

Figure S21. ^{13}C-NMR of **17a**

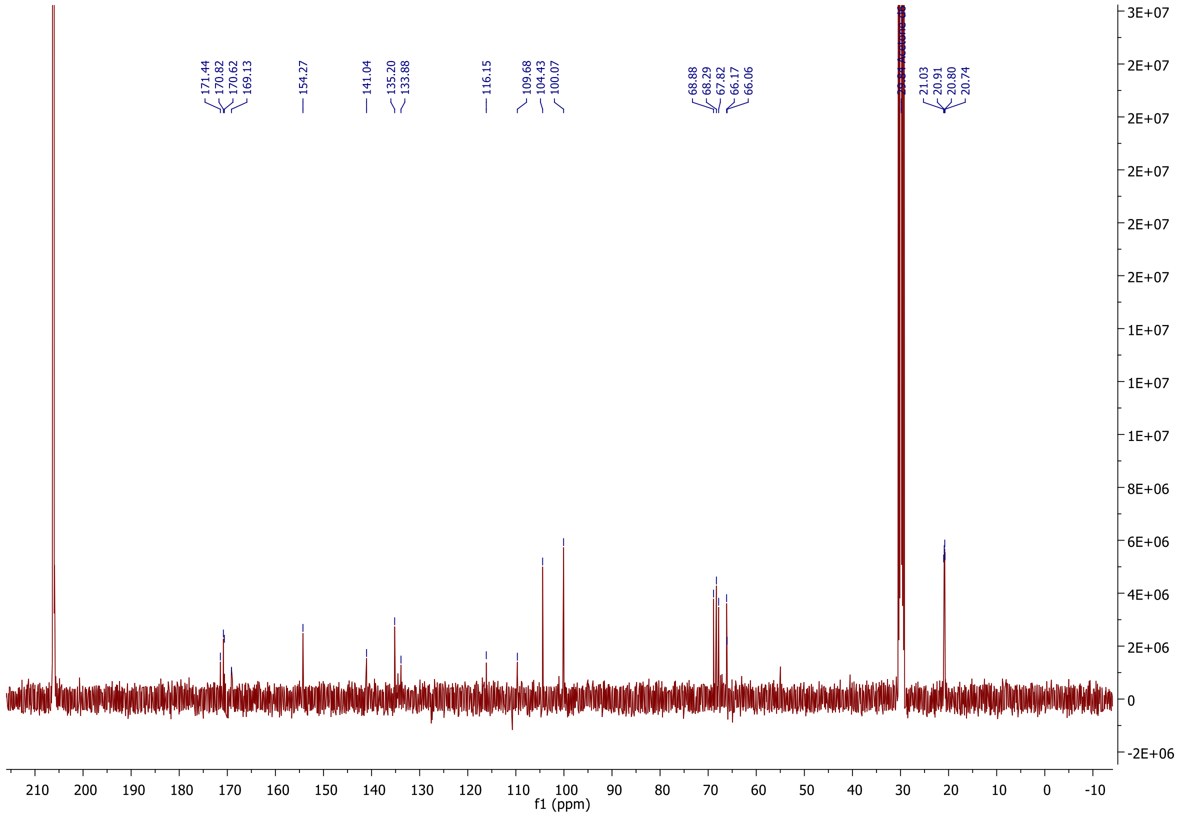

Figure S22. ^{1}H-NMR of **24**

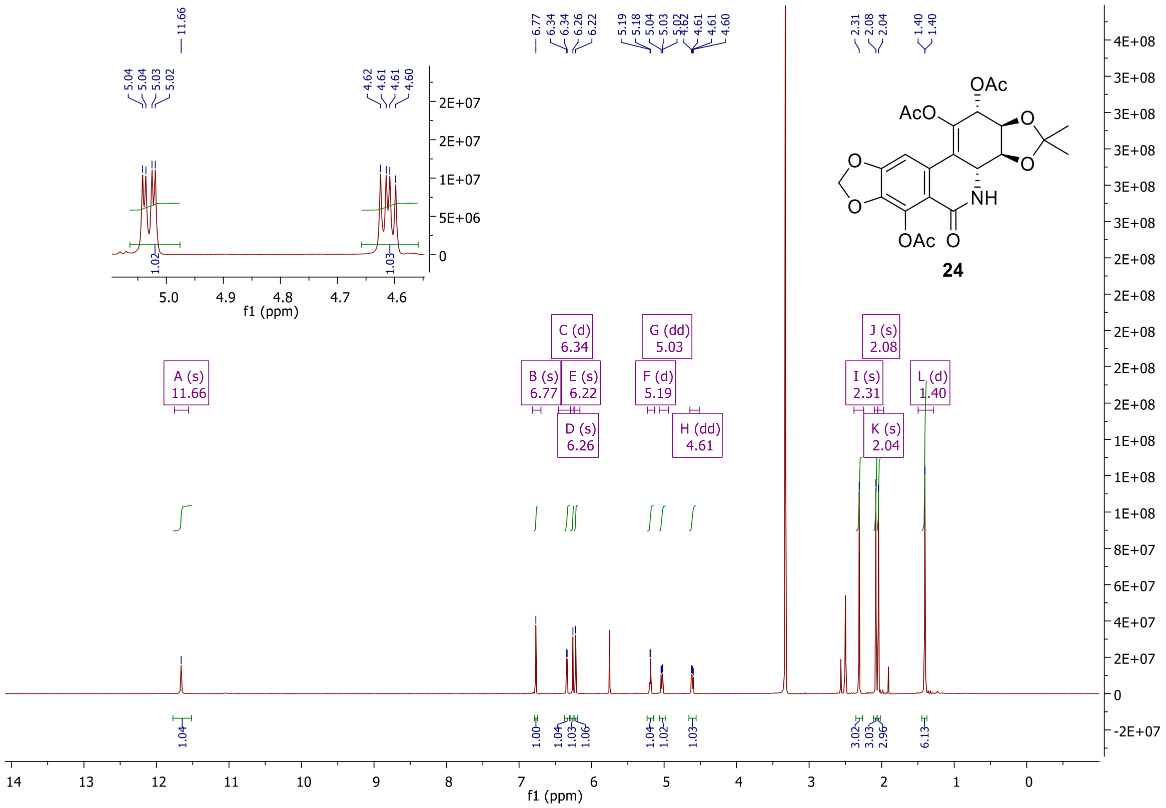

Figure S23. ^{13}C-NMR of **24**

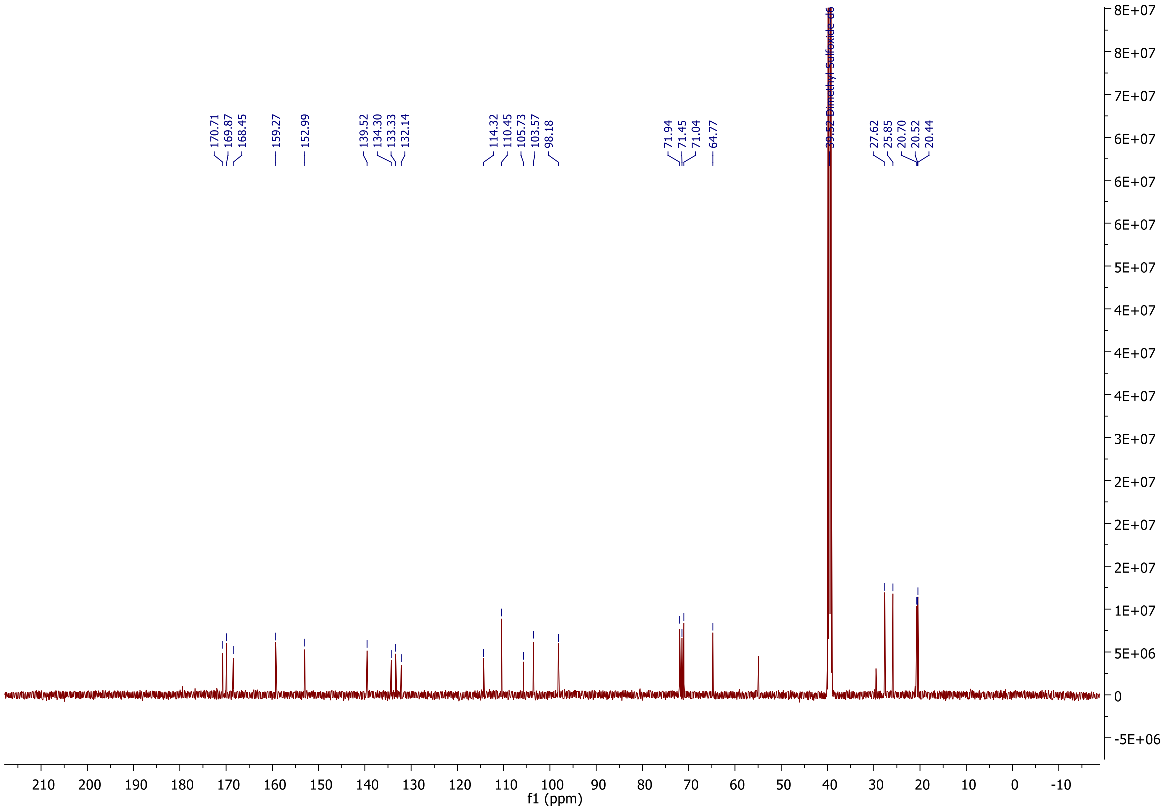

Figure S24. ^{1}H-^{15}N HSQC of **24**

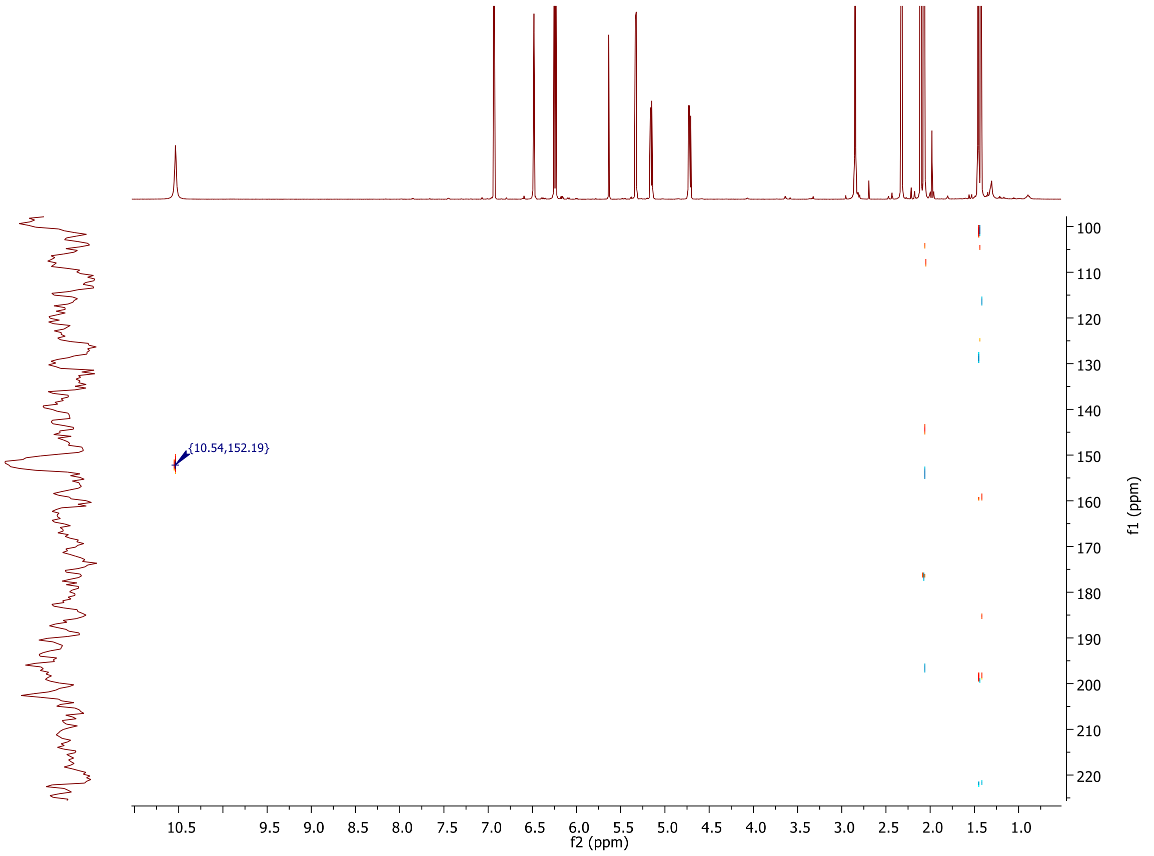

Figure S25. ^{1}H-^{15}N HMBC of **24**

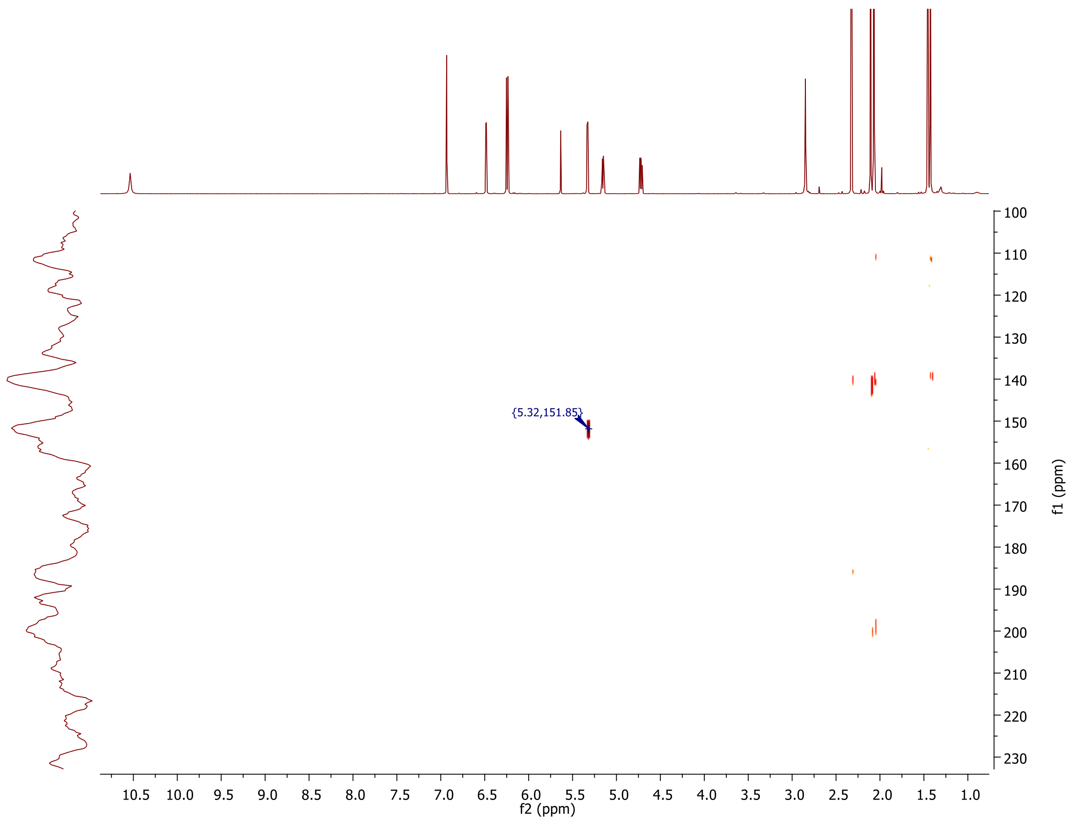

Figure S26. ^{1}H-NMR of **25**

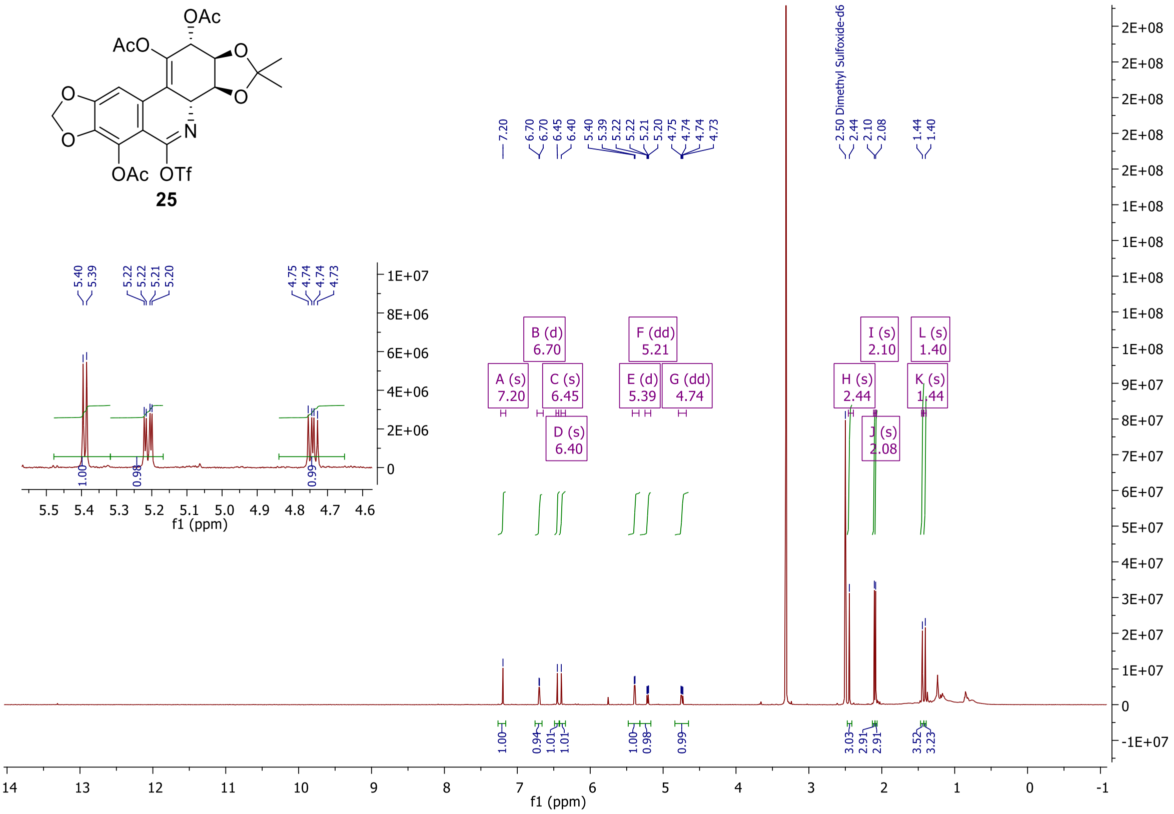

Figure S27. ^{13}C-NMR of **25**

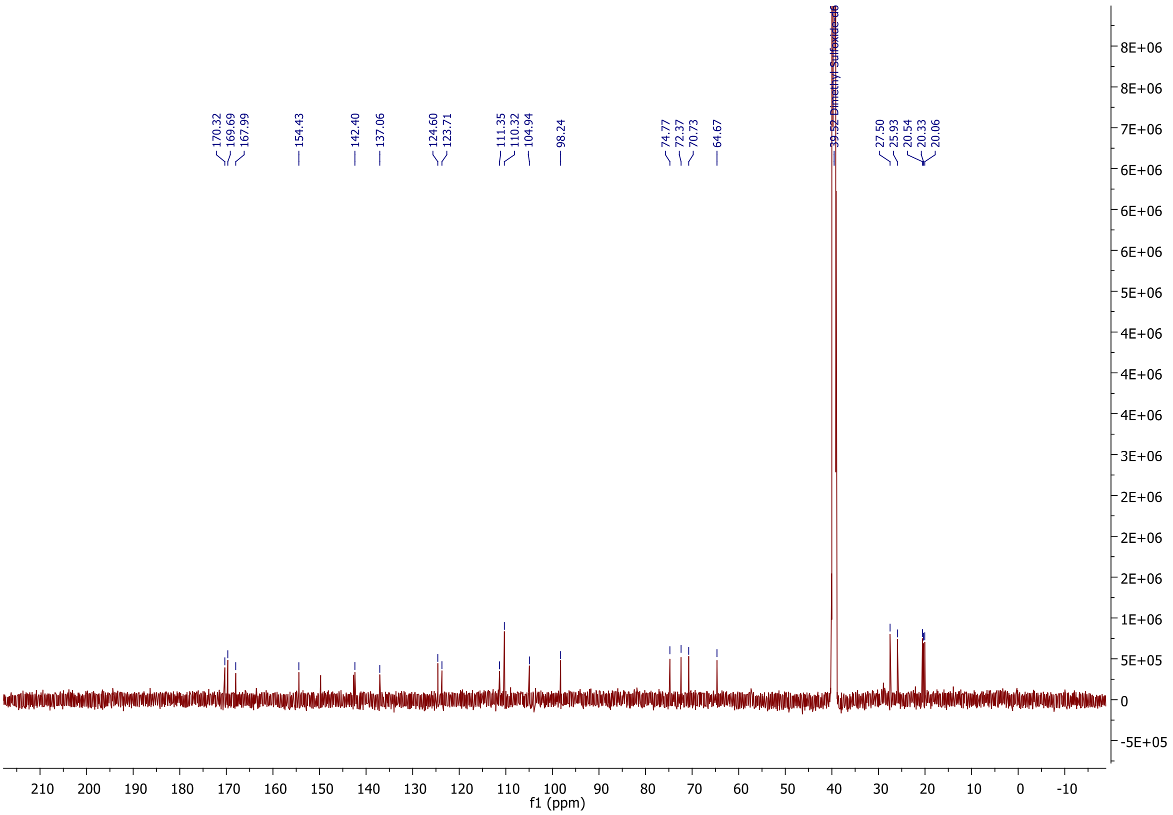

Figure S28. ^{19}F-NMR of **25**

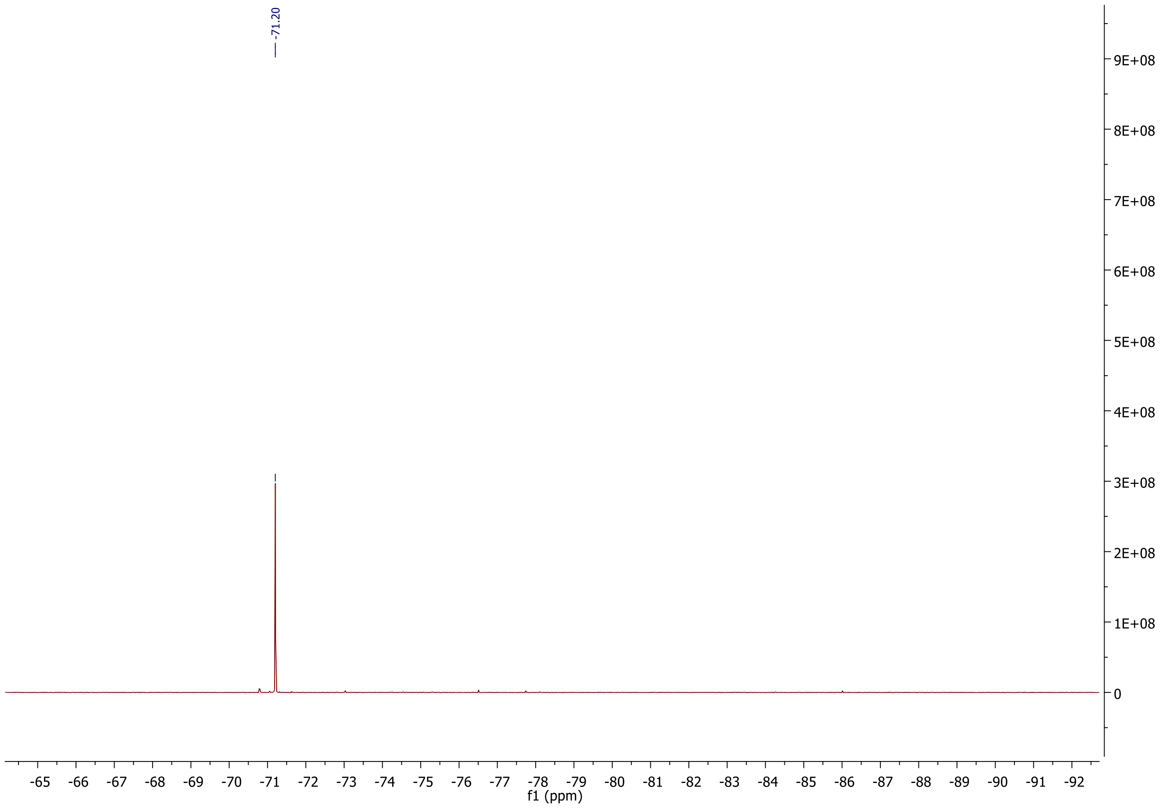

Figure S29. ^{1}H-NMR of **26**

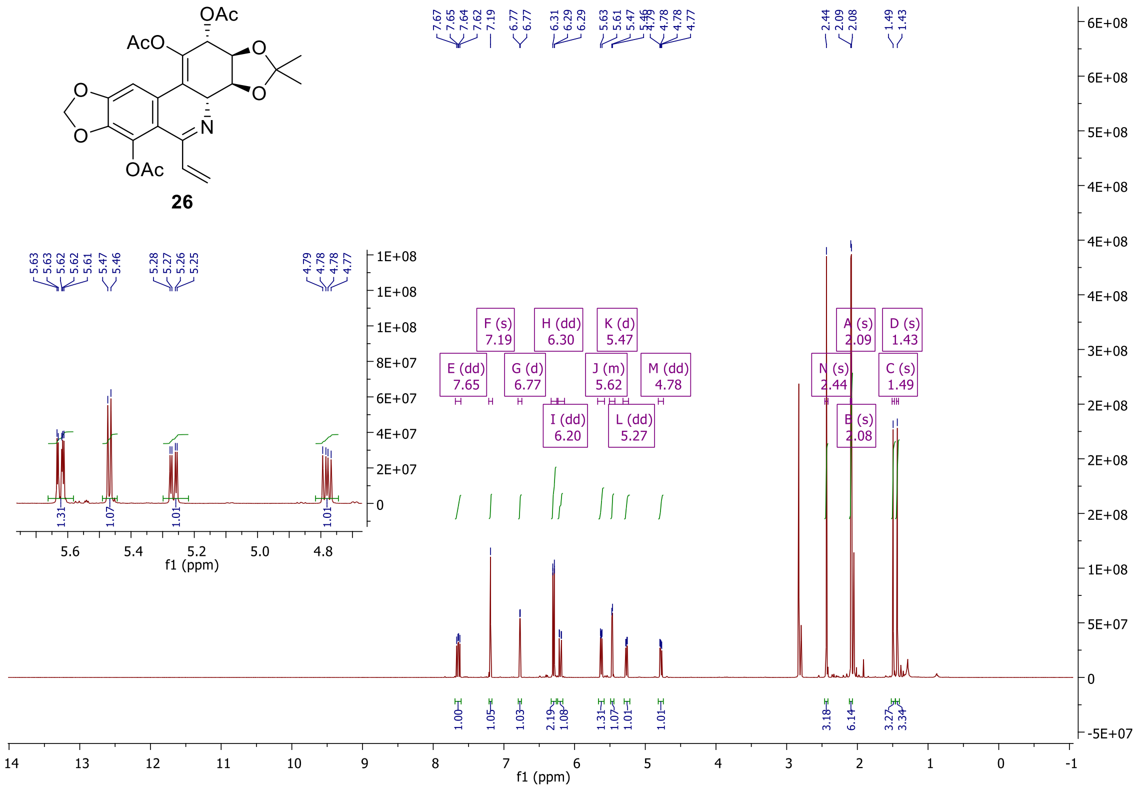

Figure S30. ^{13}C-NMR of **26**

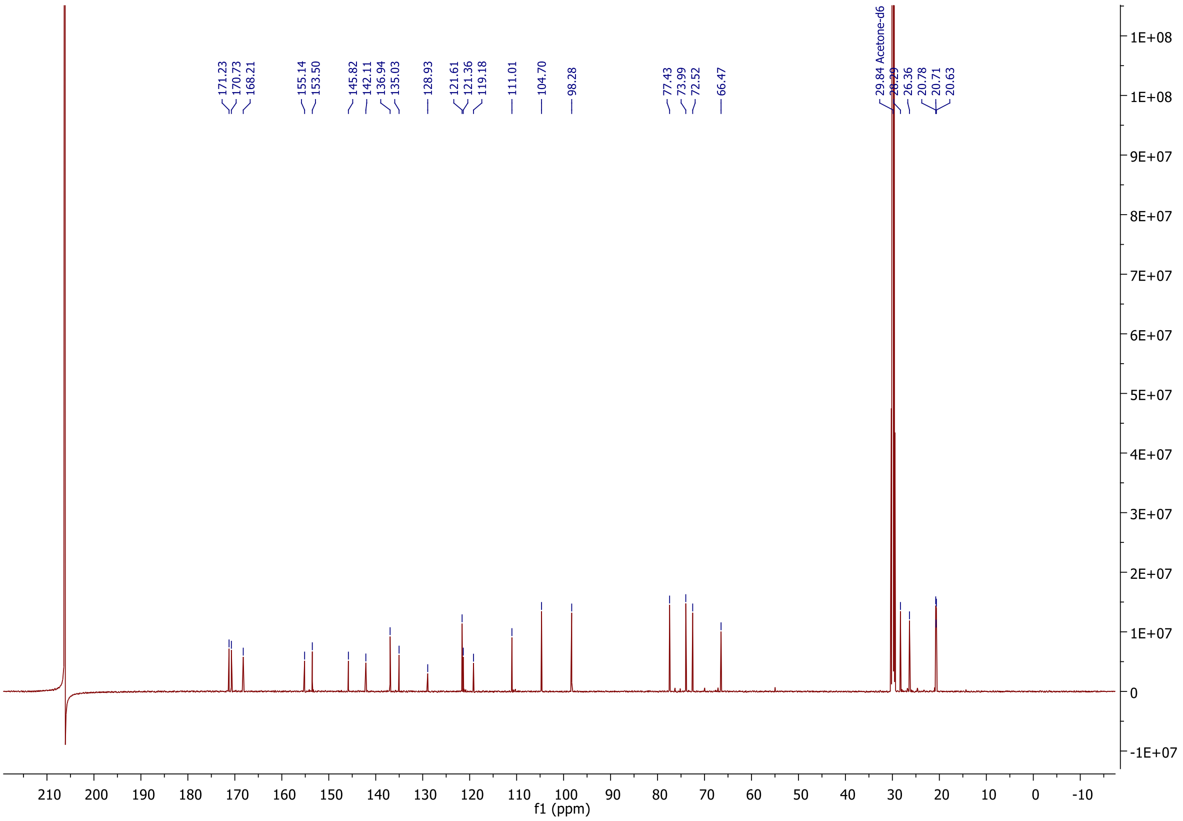

Figure S31. ^{1}H-^{15}N HMBC of **26**

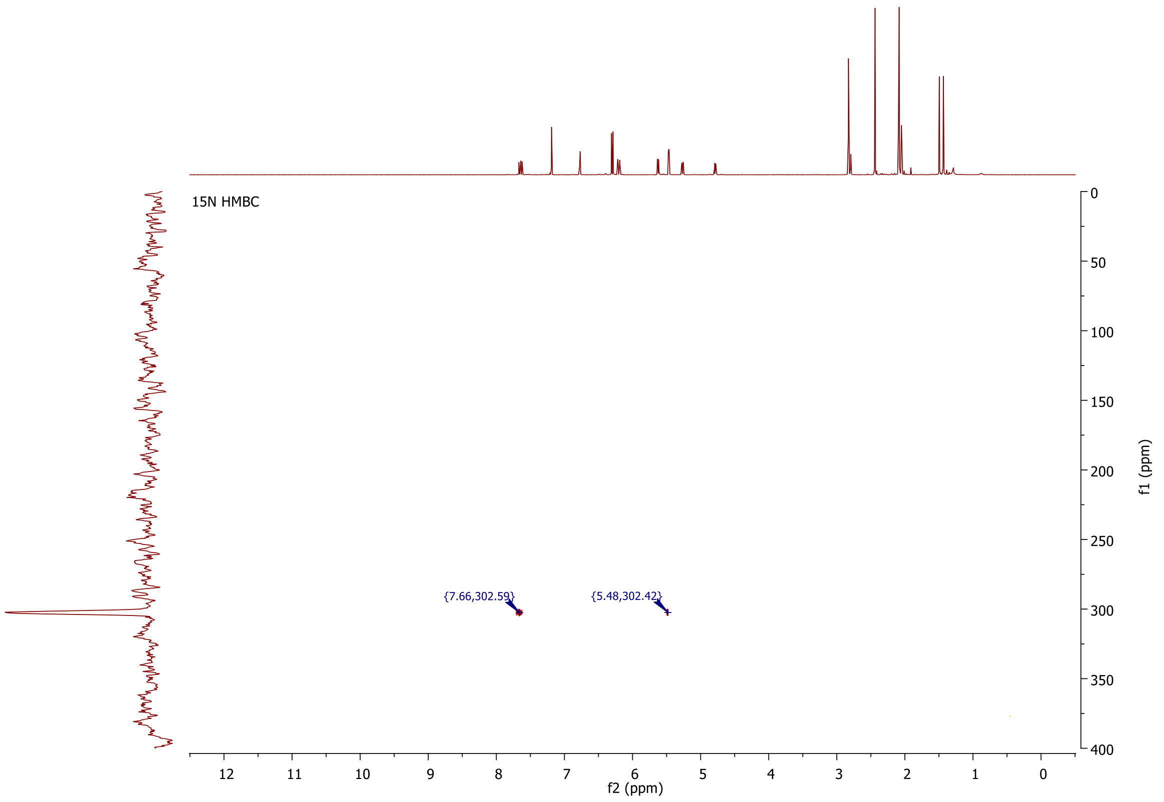

Figure S32. ^{1}H-NMR of **27**

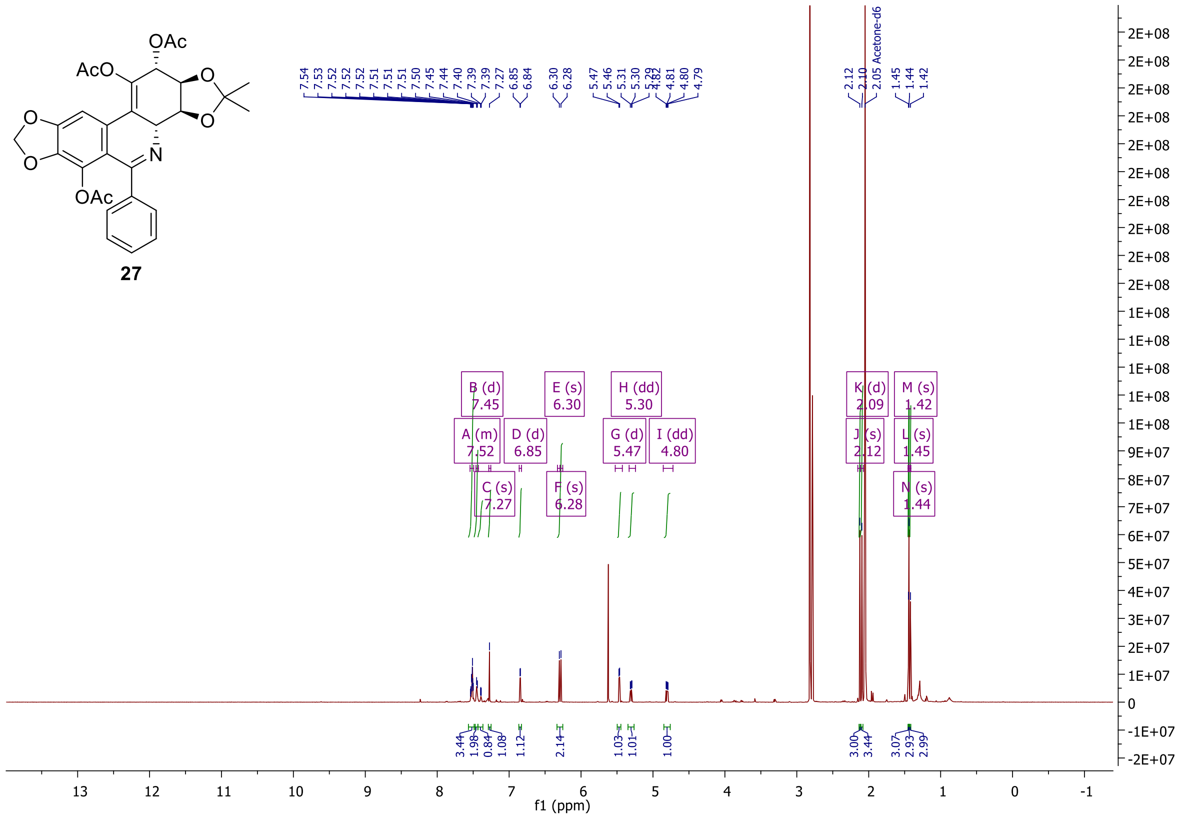

Figure S33. ^{13}C-NMR of **27**

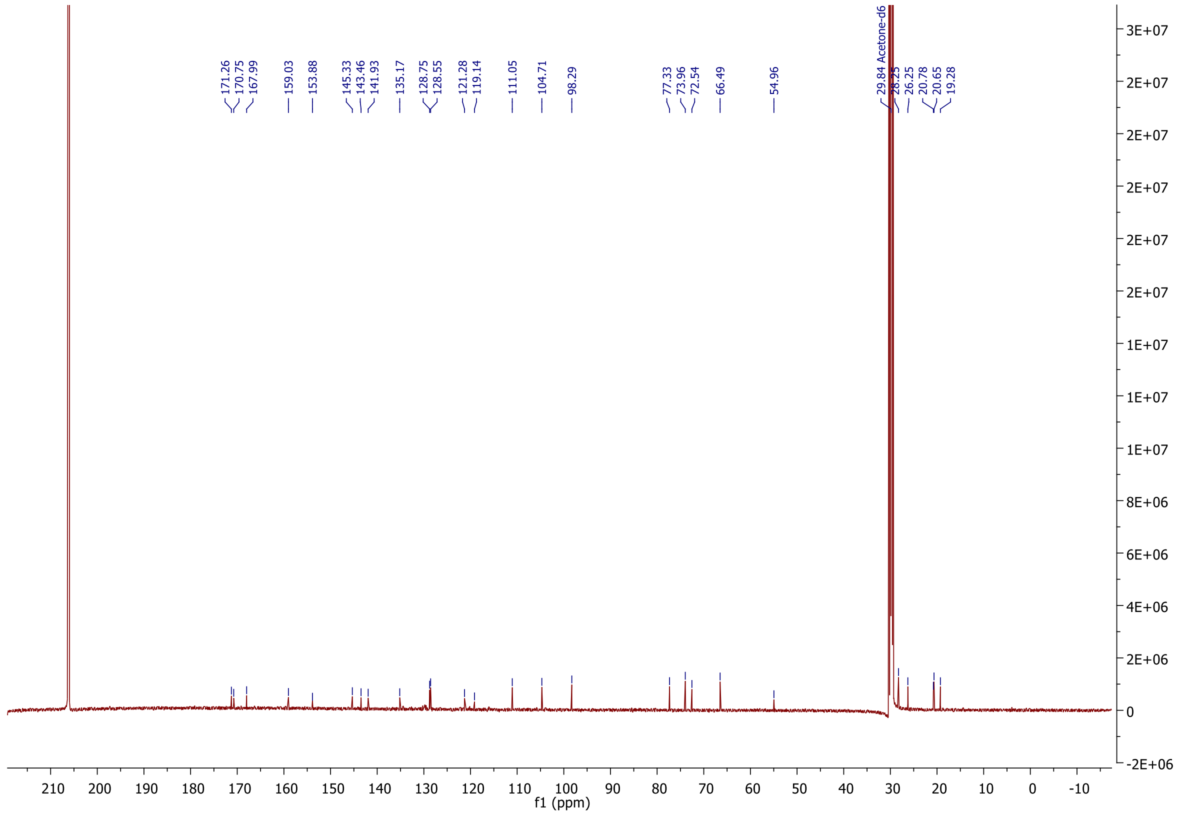

Figure S34. ^{1}H-NMR of **28**

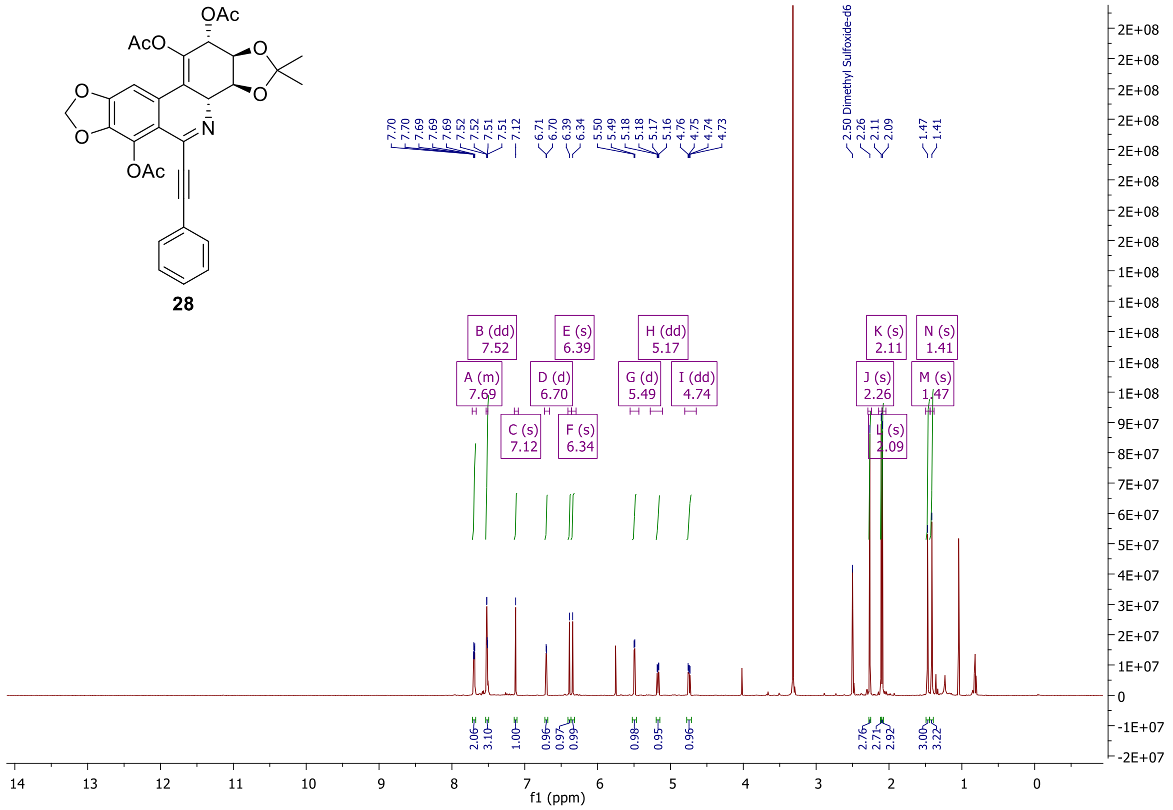

Figure S35. ^{13}C-NMR of **28**

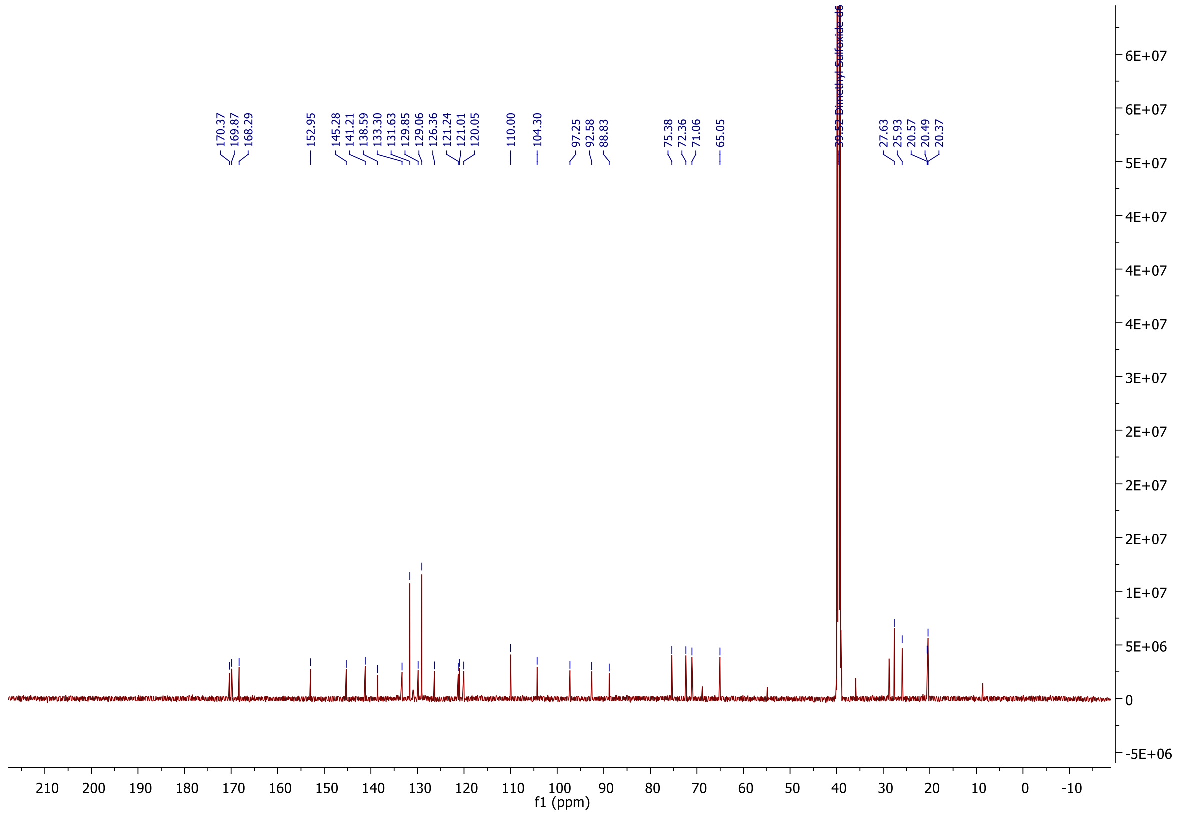

Figure S36. ^{1}H-NMR of **29**

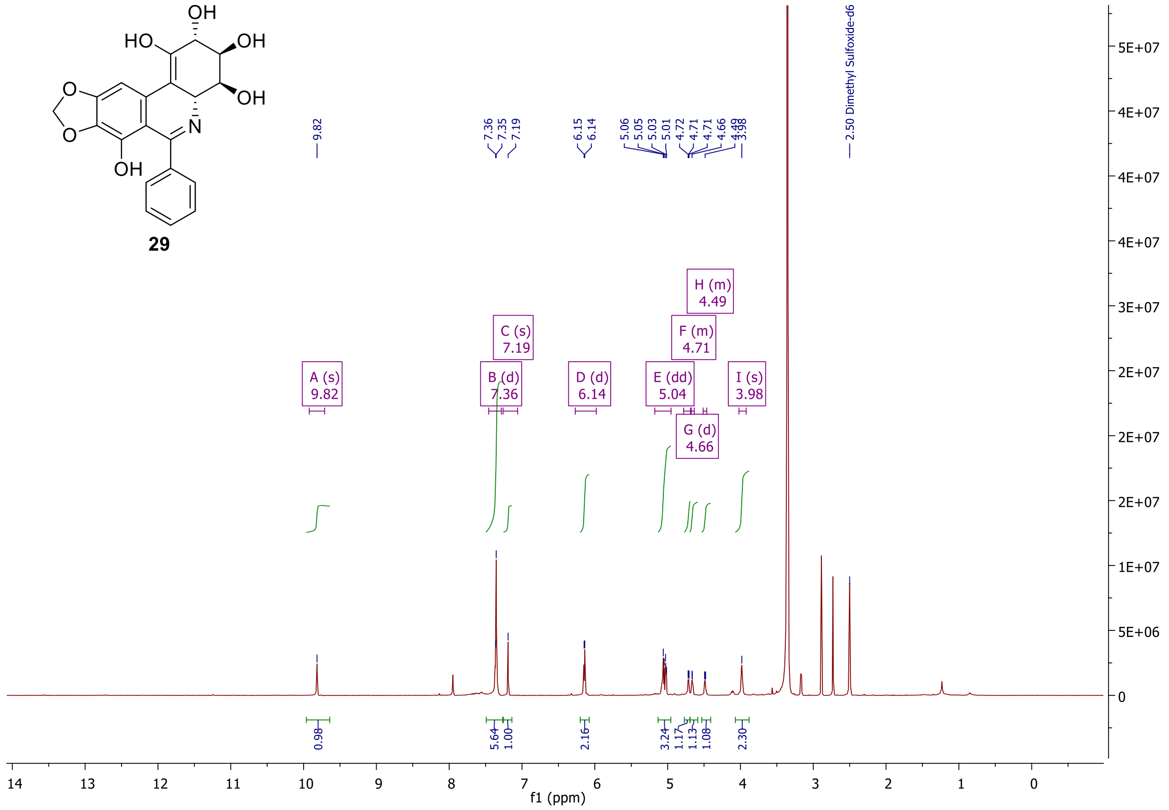

Figure S37. ^{13}C-NMR of **29**

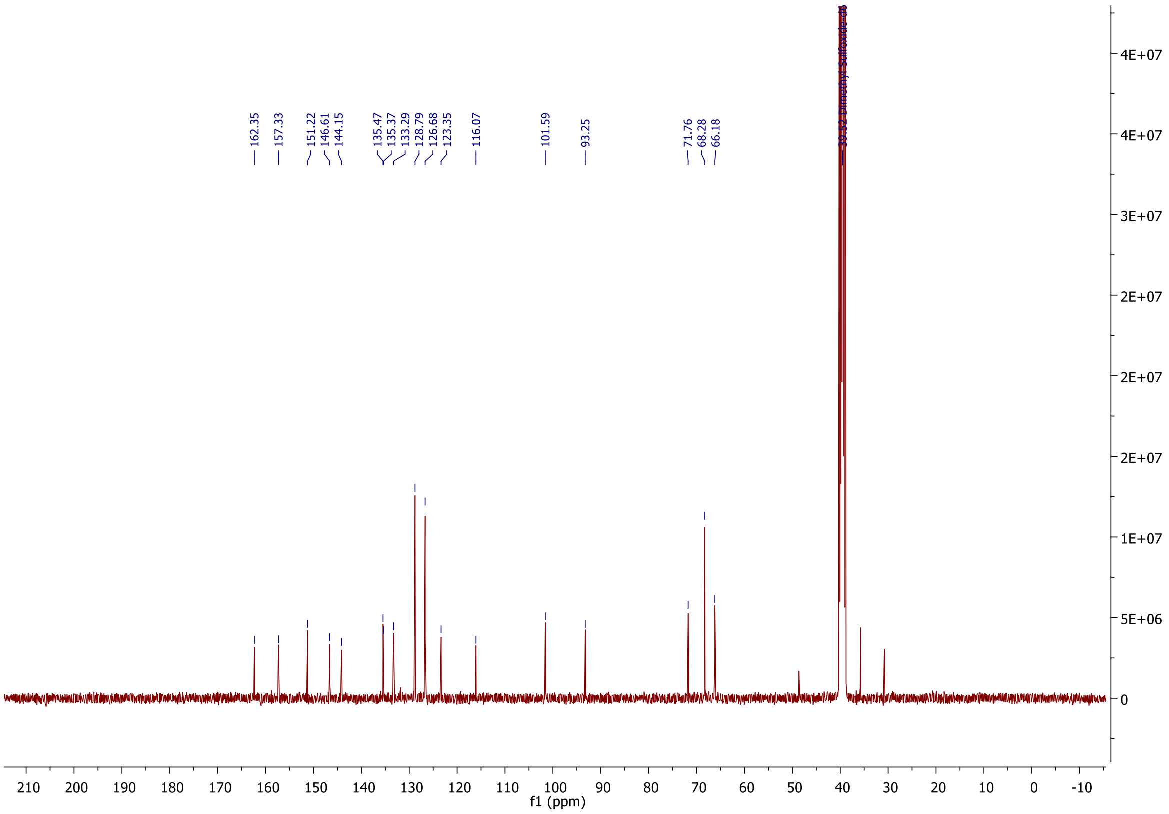

Figure S38. ^{1}H-NMR of **30**

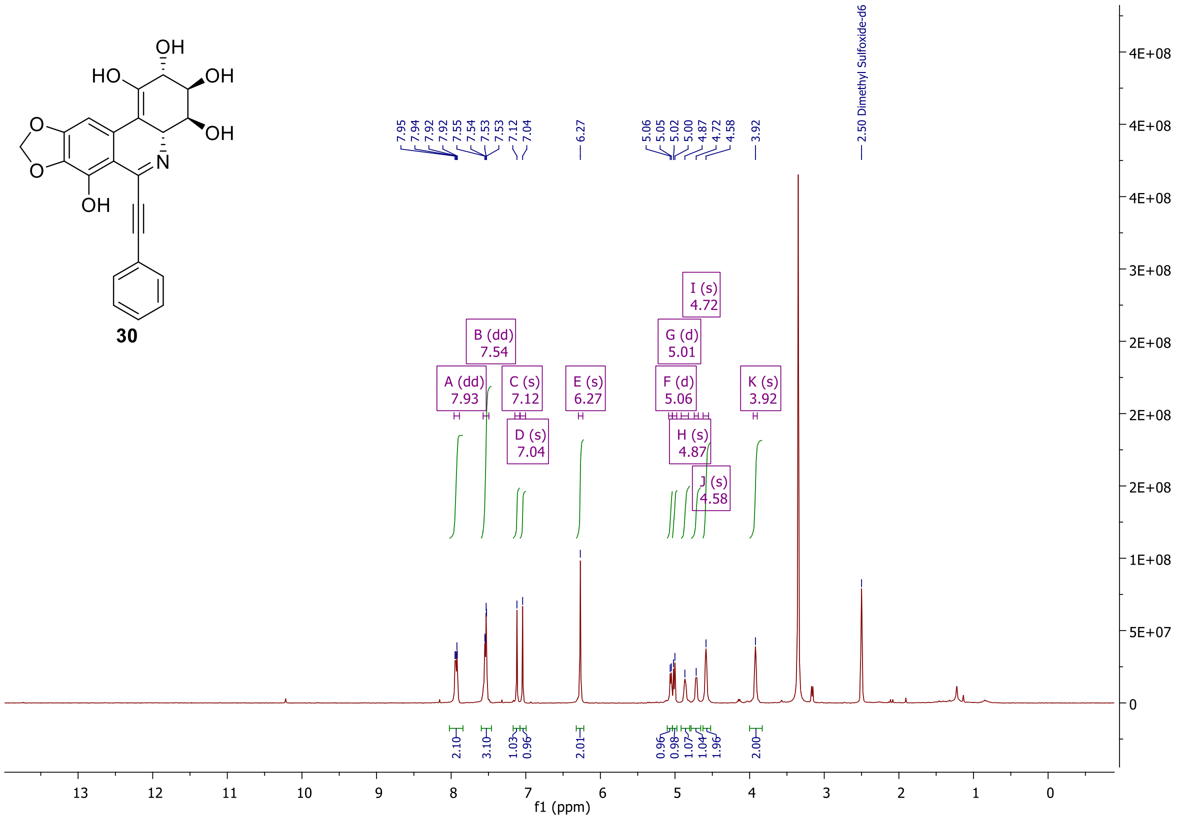

Figure S39. ^{13}C-NMR of **30**

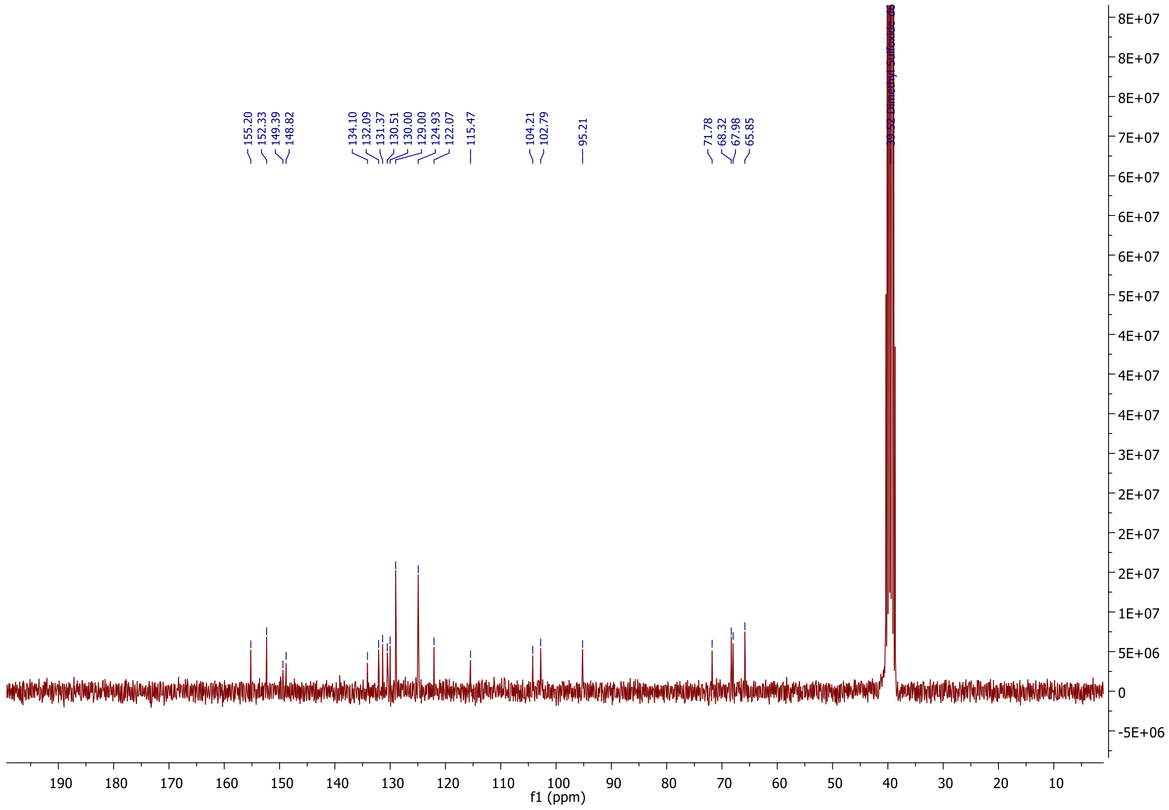

Figure S40. ^{1}H-NMR of **31**

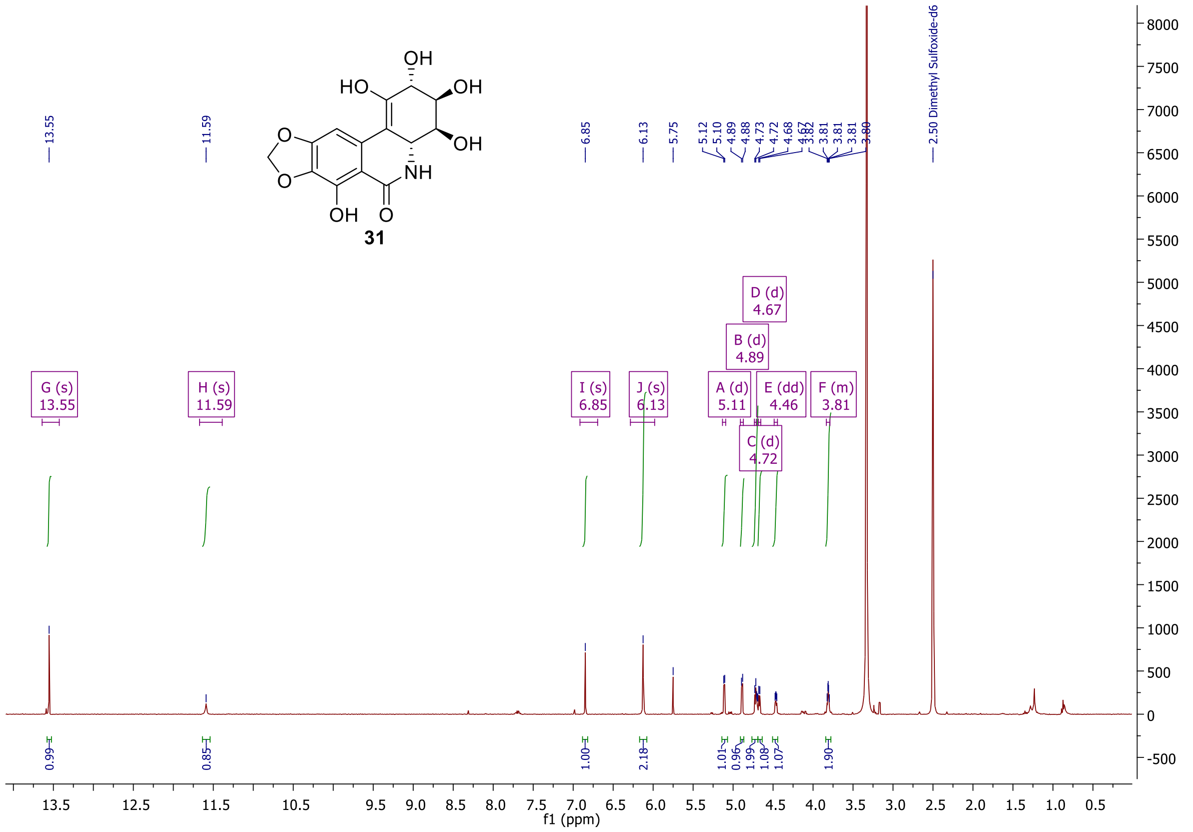

Figure S41. ^{13}C-NMR of **31**

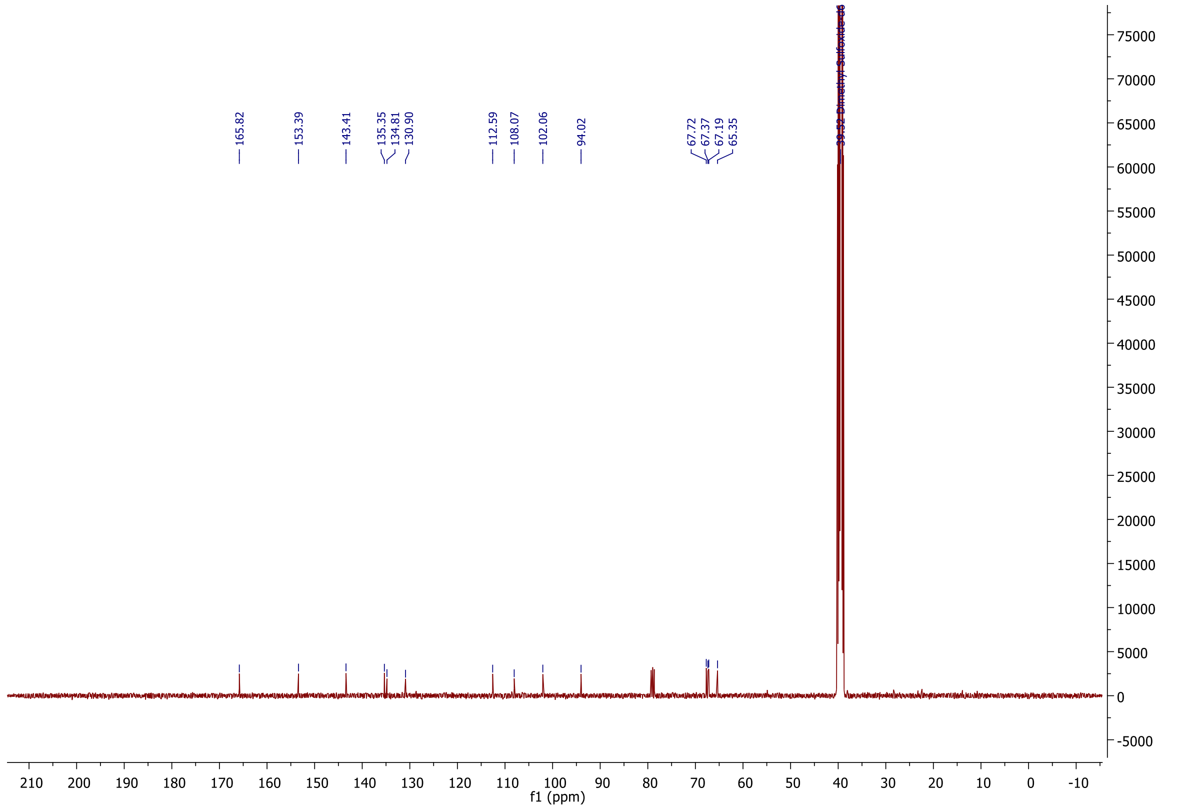

Figure S42. ^{1}H-NMR of **33**

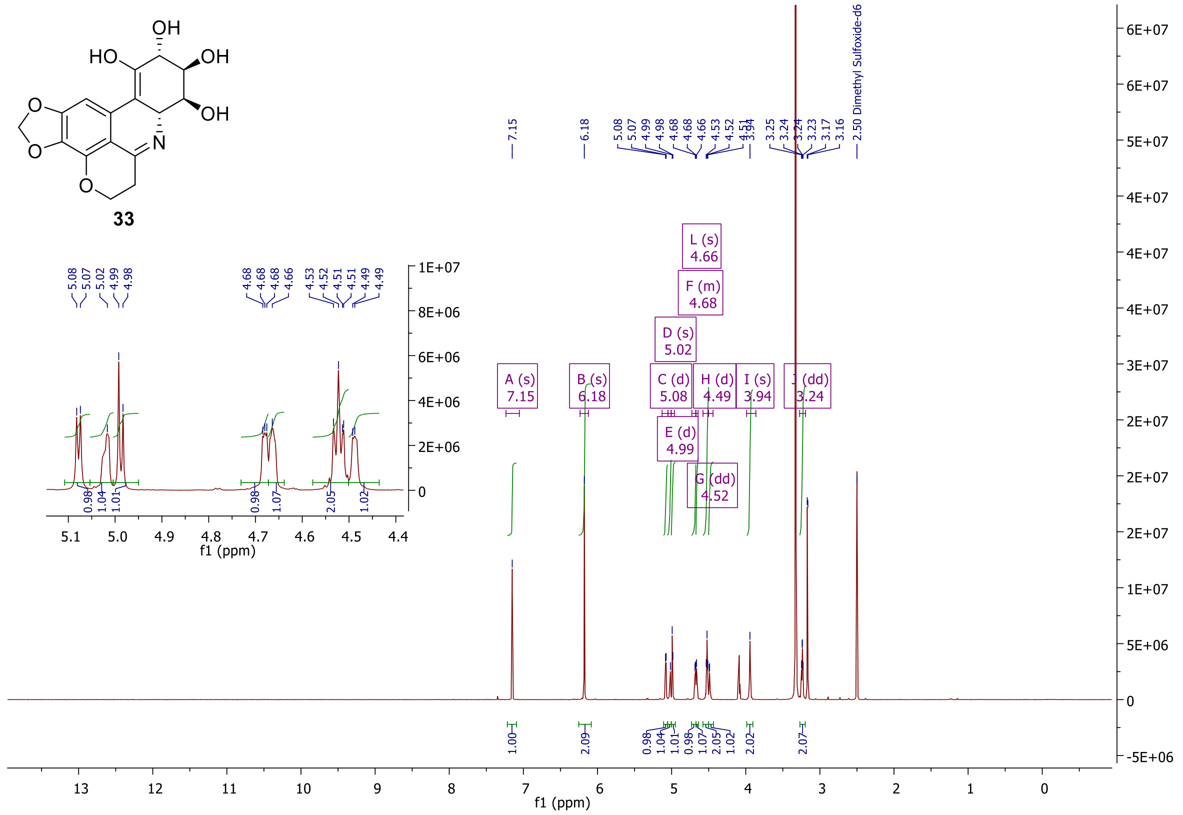

Figure S43. ^{13}C-NMR of **33**

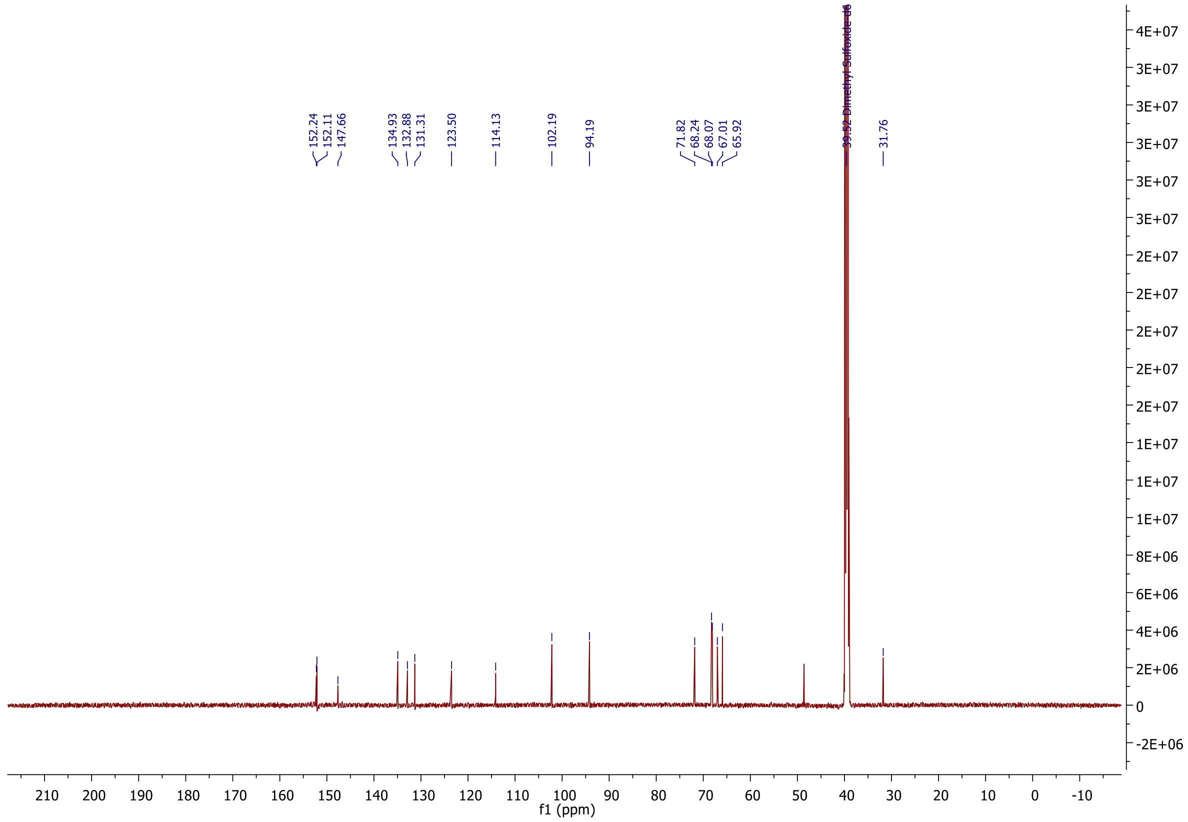

Supplement: Supplementary file 1 [file molecules-27-04141-s001.zip › molecules-1758233-supplementary.pdf]
